# Effects of Drugs Formerly Suggested for COVID-19 Repurposing on Pannexin1 Channels

**DOI:** 10.3390/ijms23105664

**Published:** 2022-05-18

**Authors:** Anne Caufriez, Andrés Tabernilla, Raf Van Campenhout, Axelle Cooreman, Kaat Leroy, Julen Sanz Serrano, Prashant Kadam, Bruna dos Santos Rodrigues, Arthur Lamouroux, Steven Ballet, Mathieu Vinken

**Affiliations:** 1 Department of Pharmaceutical and Pharmacological Sciences, Vrije Universiteit Brussel, Laarbeeklaan 103, 1090 Brussels, Belgium; anne.caufriez@vub.be (A.C.); andres.tabernilla.garcia@vub.be (A.T.); raf.van.campenhout@vub.be (R.V.C.); axelle.cooreman@vub.be (A.C.); kaat.leroy@vub.be (K.L.); julen.sanz.serrano@vub.be (J.S.S.); prashant.kadam@vub.be (P.K.); bruna.dos.santos.rodrigues@vub.be (B.d.S.R.); 2 Departments of Chemistry and Bioengineering Sciences, Vrije Universiteit Brussel, Pleinlaan 2, 1050 Brussels, Belgium; arthur.lamouroux@vub.be (A.L.); steven.ballet@vub.be (S.B.)

**Keywords:** COVID-19, antiviral and anti-inflammatory drugs, pannexin1

## Abstract

Although many efforts have been made to elucidate the pathogenesis of COVID-19, the underlying mechanisms are yet to be fully uncovered. However, it is known that a dysfunctional immune response and the accompanying uncontrollable inflammation lead to troublesome outcomes in COVID-19 patients. Pannexin1 channels are put forward as interesting drug targets for the treatment of COVID-19 due to their key role in inflammation and their link to other viral infections. In the present study, we selected a panel of drugs previously tested in clinical trials as potential candidates for the treatment of COVID-19 early on in the pandemic, including hydroxychloroquine, chloroquine, azithromycin, dexamethasone, ribavirin, remdesivir, favipiravir, lopinavir, and ritonavir. The effect of the drugs on pannexin1 channels was assessed at a functional level by means of measurement of extracellular ATP release. Immunoblot analysis and real-time quantitative reversetranscription polymerase chain reaction analysis were used to study the potential of the drugs to alter pannexin1 protein and mRNA expression levels, respectively. Favipiravir, hydroxychloroquine, lopinavir, and the combination of lopinavir with ritonavir were found to inhibit pannexin1 channel activity without affecting pannexin1 protein or mRNA levels. Thusthree new inhibitors of pannexin1 channels were identified that, though currently not being used anymore for the treatment of COVID-19 patients, could be potential drug candidates for other pannexin1-related diseases.

## 1. Introduction

The severe acute respiratory syndrome coronavirus 2 (SARS-CoV-2) has affected more than 518 million people worldwide, resulting in over 6.25 million coronavirus disease 2019 (COVID-19)-related deaths, according to the COVID-19 case tracker of the Johns Hopkins University [1]. SARS-CoV-2 infections trigger a dysfunctional immune response characterised by widespread and uncontrolled inflammation, which, in turn, leads to septic shock and multiorgan failure [2,3,4]. Inflammation is regulated by a myriad of entangled communication networks. In recent years, cellular channels composed of pannexin proteins have emerged as key players in the onset and exacerbation of inflammation [5,6]. Pannexins are transmembrane proteins that form channels connecting the cytosol to the extracellular environment. The pannexin family consists of three members (Panx1-3), of which Panx1 is the most widespread in mammalian tissues [7,8]. When opened, Panx1 channels form transmembrane conduits, allowing the passage of ions and molecules of less than 1 kilodalton (kDa), including adenosine triphosphate (ATP) [9,10,11]. Panx1 channels have been broadly linked to cell death [9,11] and inflammatory processes [5,6,12,13,14]. More specifically, Panx1 channels contribute to inflammatory responses by facilitating cleavage of pro-caspase1 in the NACHT-, LRR-, and pyrin-domain-containing protein 3 (NLRP3) inflammasome, a multiprotein complex that activates interleukin 1β and interleukin 18 [12,15,16]. In addition, Panx1 channel-mediated ATP release leads to an upregulation of Panx1 phosphorylation and of vascular cell adhesion molecule 1 and is, therefore, involved in the activation and migration of leukocytes [17]. Due to their contribution to inflammation and the link to viral infectious diseases, such as human immunodeficiency virus (HIV) infections and hepatitis C, it seems likely to assume that Panx1 channels can also play a role in COVID-19 [18,19,20,21]. Recently, it was found that the SARS-CoV-2 spike protein, a fusion protein essential in the induction of the infection, triggers prolonged Panx1 channel opening [18]. Furthermore, Panx1 mRNA and protein expression levels were shown to be elevated in samples of patients with a SARS-CoV2 infection, indicating a role of Panx1 channel opening in COVID-19 and suggesting Panx1 channels as potential drug targets [18]. Several drugs have been proposed for the treatment of COVID-19 due to their antiviral and/or anti-inflammatory effects, including hydroxychloroquine, chloroquine, azithromycin, dexamethasone, ribavirin, remdesivir, favipiravir, lopinavir and the combination of the latter with ritonavir (Table 1) [22,23]. In the present study, the effects of these drugs on Panx1 channels were investigated at the transcriptional, translational, and functional level.

## 2. Results

### 2.1. Determination of Working Concentrations of the Drug Panel

**Figure 1:**
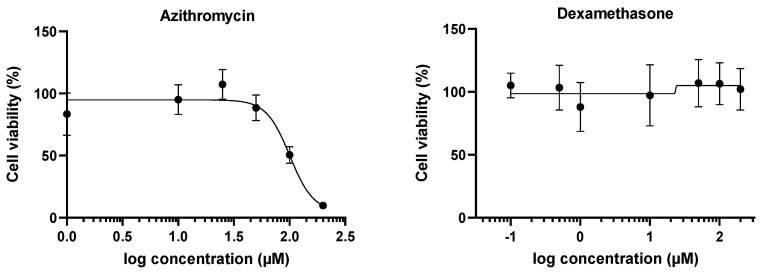

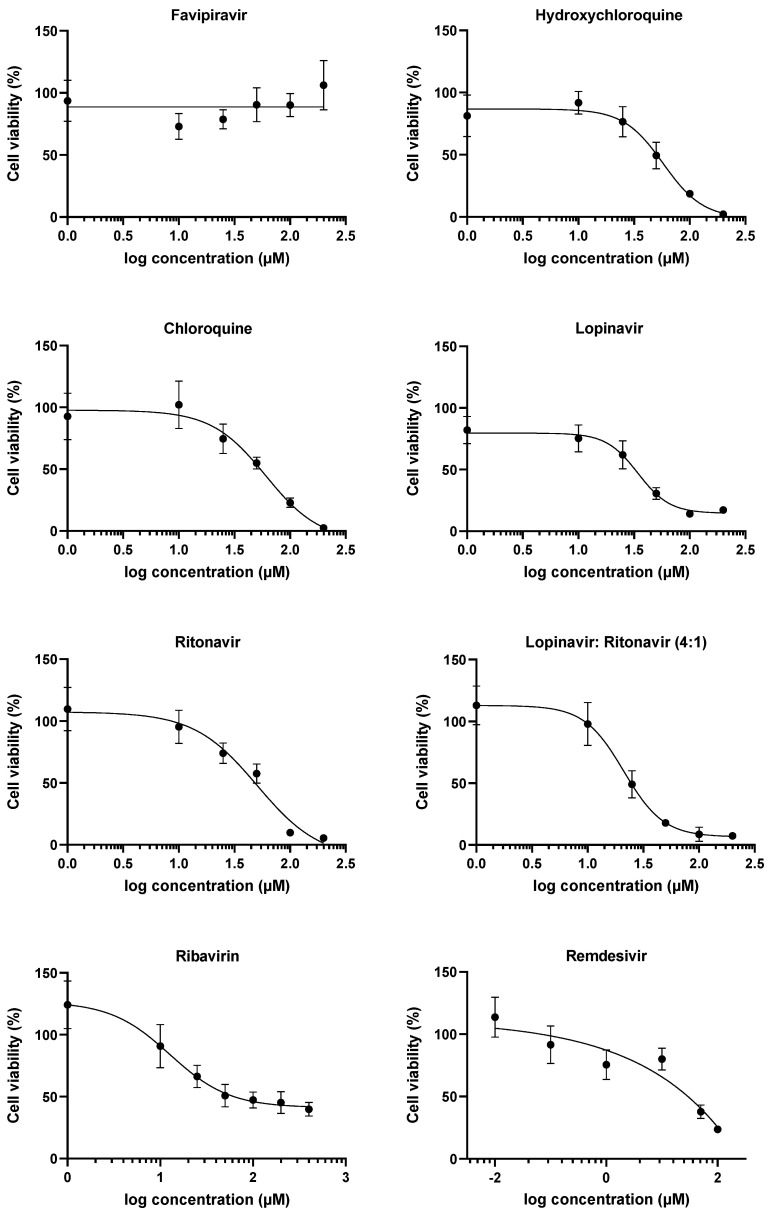
Cell viability curves for the determination of the CC_10_ after 24 h exposure of transduced Dubca cells overexpressing human Panx1 to the drug panel. A sigmoidal curve was fitted by means of non-linear regression using GraphPad^®^ Prism to determine the CC_10_ value. Data are expressed as mean ± standard deviation (*N* = 3, *n* = 4) and visualised in separate graphs per drug.

The majority of the drugs evaluated in this study are anti-inflammatory drugs or antiviral drugs able to influence the inflammatory process in an indirect way [23]. Ritonavir categorises as the latter but was not directly implicated in the treatment of COVID-19 patients [33]. Ritonavir is, however, well-known to increase the bioavailability of other human immunodeficiency virus protease inhibitors, such as lopinavir. The combination of these drugs in a 4:1 ratio was administered to patients in COVID-19 trials [34,35] and, therefore, was included as such in the present study. To determine appropriate working concentrations for each of the nine drugs and/or their combinations, a cell viability assay was performed after 24 h of exposure to transduced Dubca cells overexpressing human Panx1. Initial test concentrations for the determination of the concentration inducing cell death in 10% of the cell population (CC_10_) were retrieved from published data (Table 1) [36,37,38,39,40]. A sigmoidal curve was fitted by means of non-linear regression to the obtained data set using GraphPad^®^ Prism (Figure 1). Based on these curves, the CC_10_ was defined for each drug. Although cytotoxicity should preferably be avoided, bulk cytotoxicity after adding compounds directly to cell cultures cannot be prevented. Hence, CC_10_ concentrations for each drug were used as a starting point in the present study [41]. For those drugs for which the CC_10_ value could not be determined in the first run of cell viability experiments, a larger concentration range was tested. Despite this second run of experiments, the CC_10_ could not be obtained for remdesivir, dexamethasone, and favipiravir, as the cytotoxic effect of these compounds was not extensive enough in a relevant concentration range to obtain a sigmoidal curve. In these cases, the maximum plasma concentration (C_max_) or 10-fold of the C_max_ was used to ensure a physiologically relevant concentration range [27,28,29]. Thereafter, the CC_10_, C_max_, or C_max_ x10 of each drug (Table 1) was used as a benchmark concentration (BC) to set a working concentration range to be tested—namely, BC-BC/2-BC/10 for protein and mRNA quantification measurements, and BCx10-BCx5-BCx2-BC-BC/2-BC/10 for the assessment of effects on Panx1 channel activity (Table 1). The shorter time of exposure (45 min), compared with the 24 h exposure window used to determine the CC_10_, allowed higher concentrations to be tested in the Panx1 channel activity assay. The broader concentration ranges of the drugs used in the Panx1 channel activity assay were additionally evaluated in a cell viability assay after 45 min of exposure. Concentrations were categorised as cytotoxic when a decrease in cell viability of more than 20% was observed in comparison with the untreated cells [42]. With the exception of the highest concentration of azithromycin (590 µM; 74.04% ± 3.38%), no cytotoxicity was observed by any of the drugs in the broader concentration range (Figure S1). Results obtained for the highest azithromycin concentration should be interpreted with caution as extracellular ATP release is both the read-out of the channel activity assay and a marker of cell death [43].

### 2.2. Effects of the Drug Panel on Panx1 Channel Activity

**Figure 2:**
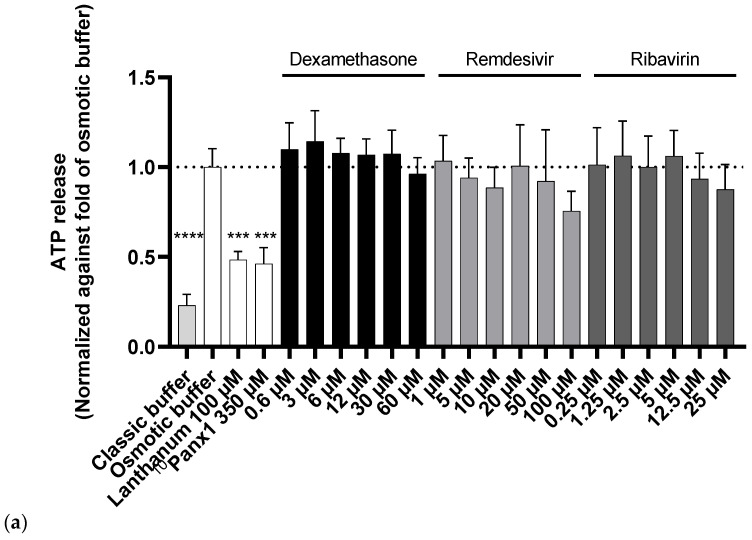

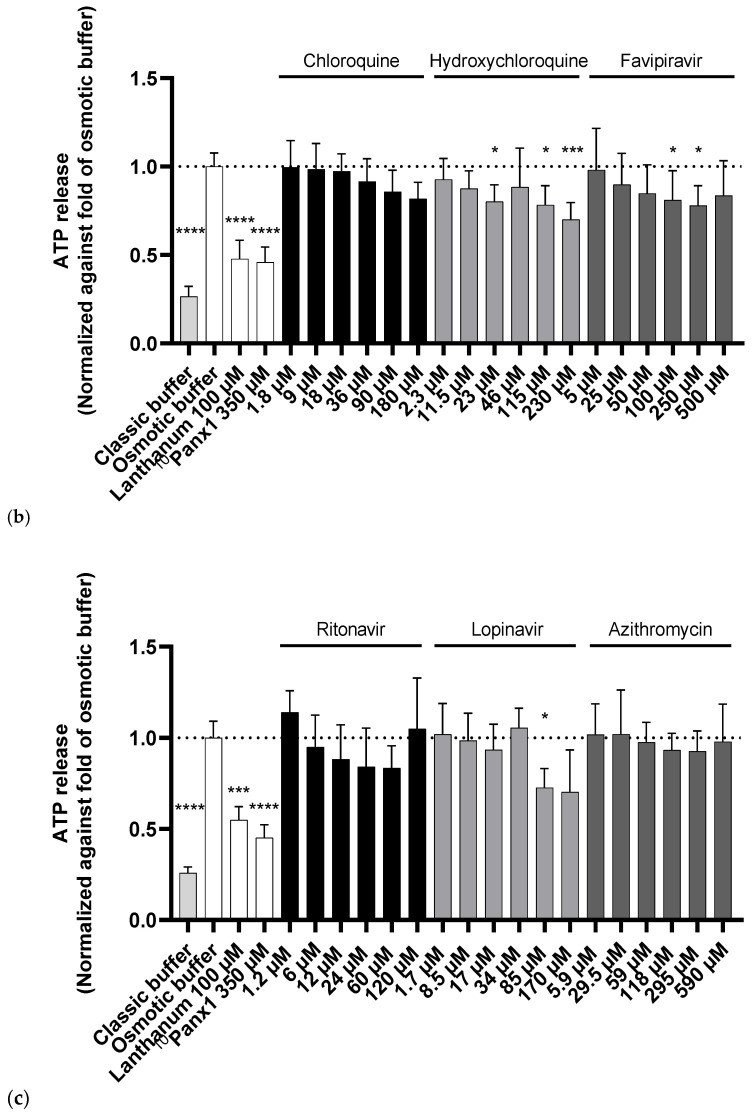

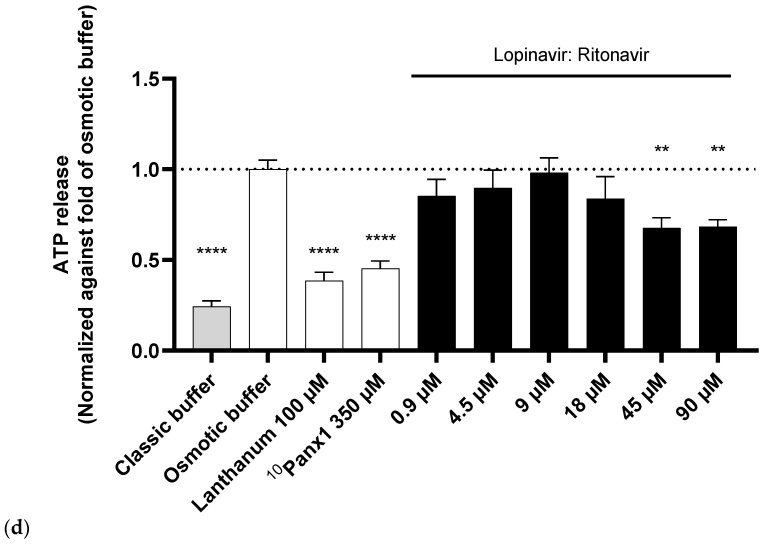
Inhibitory capacity of the drug panel in a Panx1 channel activity assay using transduced Dubca cells overexpressing human Panx1. Panx1 channel opening was triggered via osmotic shock. Extracellular ATP release was measured using a bioluminescence assay and normalised against the osmotic buffer condition. Lanthanum and ^10^Panx1 were included as positive controls. Statistical analysis was performed using a non-parametric Kruskal–Wallis test in combination with a Dunn’s test in comparison with the osmotic buffer condition. Data are expressed as mean ± standard deviation, with * *p* ≤ 0.05; ** *p* ≤ 0.0; *** *p* ≤ 0.001 and **** *p* ≤ 0.0001 (*N* = 3, *n* = 4). (**a**) dexamethasone, remdesivir, ribavirin; (**b**) chloroquine, hydroxychloroquine, favipiravir; (**c**) ritonavir, lopinavir, azithromycin; (**d**) lopinavir: ritonavir in a 4:1 ratio (concentration shown as the final concentration of lopinavir).

Panx1 channels are indispensable in inflammation [5,6,12,15,17,44]. Their channel opening is triggered by a number of stimuli, including high extracellular potassium levels. ATP released through these open Panx1 channels in the extracellular environment binds to purinergic P2 receptors, which drive the activation of NLRP3 inflammasomes [44,45,46,47]. Furthermore, Panx1-mediated ATP release has been associated with viral infections, replication, and pathogeneses [20,21,48,49]. It was previously shown that anti-inflammatory drugs [50,51] and antiviral drugs [52] are able to alter Panx1 channel activity. Accordingly, several of the drugs previously or currently proposed for COVID-19 treatment could affect Panx1 channels. To verify this hypothesis in the present study, transduced Dubca cells overexpressing human Panx1 were exposed for 45 min to six concentrations of each drug ranging from the benchmark concentration divided by ten to a tenfold the benchmark concentration (Table 1). First, cells were preincubated with the drugs for a total of 15 min. This preincubation phase was followed by the forced opening of Panx1 channels using an osmotic shock through the application of an elevated concentration of extracellular potassium in parallel with an additional 30 min of exposure to the drugs. Extracellular ATP was measured as an indicator of Panx1 channel opening, whereby ^10^Panx1 and lanthanum, two well-known Panx1 channel inhibitors, were included as positive controls (Figure 2 and Figure S2) [51,53,54,55]. A significant decrease in extracellular ATP release was observed for three out of the nine drugs—namely, favipiravir, hydroxychloroquine sulphate, and lopinavir (Figure 2). Hydroxychloroquine was the most potent drug in this regard, inhibiting channel activity already at 23 µM (Figure 2b), while significant inhibition was only seen in the higher concentration ranges of favipiravir (100 µM and 250 µM) (Figure 2b) and lopinavir (85 µM) (Figure 2c). Ritonavir did not decrease extracellular ATP release; however, the combination of ritonavir and lopinavir (45 µM and 90 µM) maintained the potency of the latter to alter Panx1 channel activity (Figure 2c,d). Chloroquine, a structural analogue of hydroxychloroquine, showed a similar lowering effect on extracellular ATP release, albeit not significantly (Figure 2b). A comparable concentration-dependent trend was seen for remdesivir (Figure 2a).

### 2.3. Effects of the Drug Panel on Panx1 Protein Expression

**Figure 3:**
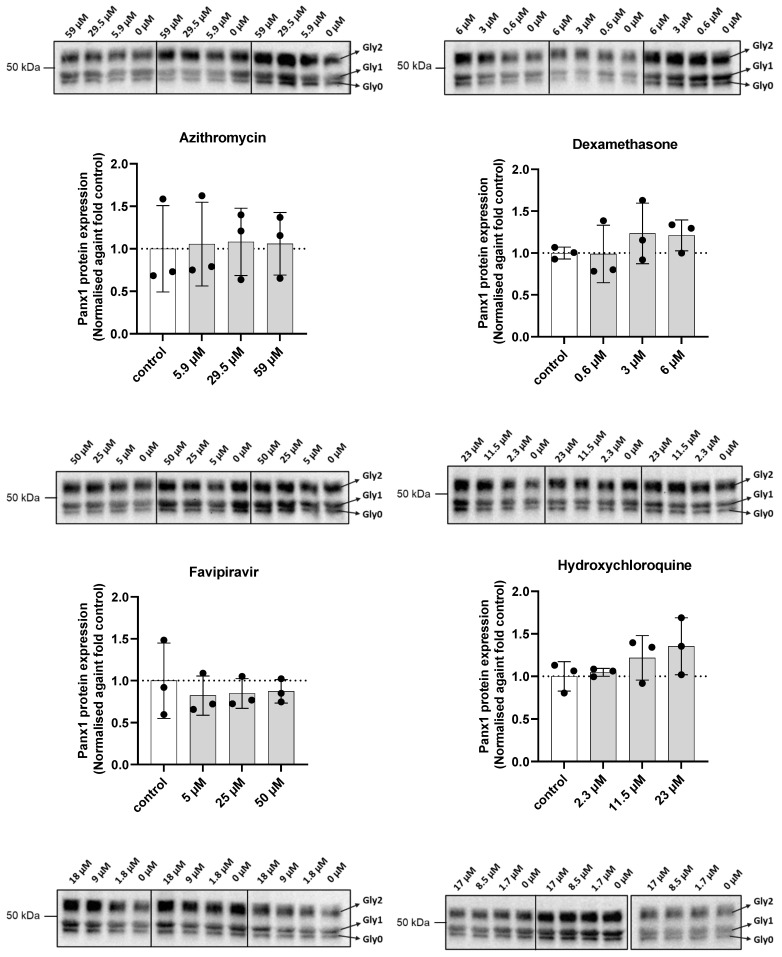

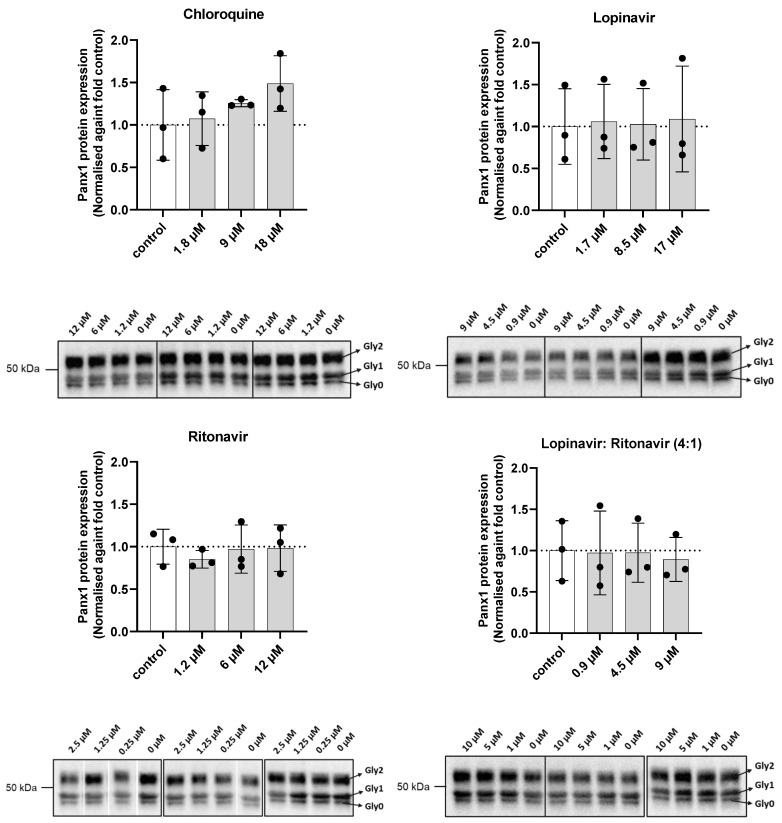

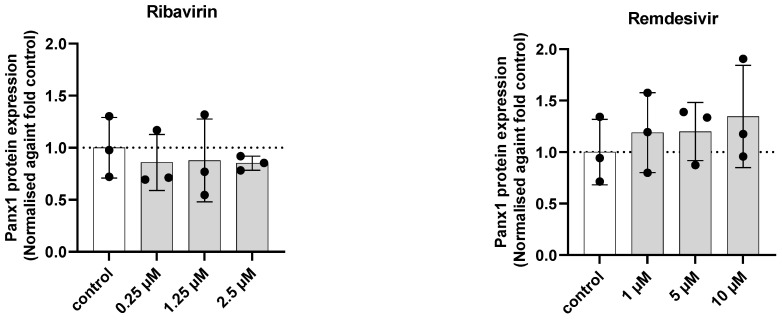
Panx1 protein expression after 24 h exposure of transduced Dubca cells overexpressing human Panx1 to the drug panel. Immunoblotting was performed, and data were extracted from the obtained blots using Image Lab 6.1 software. Data were normalised against total protein loading and the respective controls. Statistical analysis was performed using a parametric one-way ANOVA or non-parametric Kruskal–Wallis test in combination with a Dunnett’s or Dunn’s test, depending on the normality of the data distribution. The respective biological replicates are represented by separate dots in the graphs and by separate frames on the blot images (*N*). Data are expressed as mean ± standard deviation (*N* = 3, *n* = 1) and visualised in separate graphs per drug with their respective blot images on top.

Panx1 protein expression is upregulated under inflammatory conditions [56,57] and upon SARS-CoV-2 infection [18]. As anti-inflammatory drugs have shown to alter Panx1 expression, several of the compounds in the drug panel were anticipated to affect protein expression as well [23,58]. In the present study, transduced Dubca cells overexpressing human Panx1 were exposed for 24 h to the drugs at concentrations equal to and lower than the benchmark concentration (Table 1). Protein extraction was followed by semi-quantitative immunoblot analysis and relative protein levels were obtained after normalisation against total protein loading (Figure S3). This normalisation procedure provides a more robust method in comparison with the use of loading controls in the form of proteins encoded by housekeeping genes [59]. Panx1 proteins were detected at a molecular weight of around 50 kDa as a three-band signal, representing the non-glycosylated (Gly0), high-mannose (Gly1), and complex glycosylated variants (Gly2) (Figure 3) [60,61,62]. The effect of the drug panel on both total Panx1 protein levels (Figure 3), as well as on the individual glycosylated isoforms (Figure S4), was evaluated, as the glycosylation state of Panx1 proteins determines their cellular localisation and, therefore, channel activity. The Gly2 isoform, in specific, was of interest, as it is abundantly expressed at the cell membrane [60,61,62,63,64]. For the purpose of quantifying these different isoforms, the ratios between the different glycosylated and non-glycosylated variants were assessed (Figure S4). However, none of the drugs in any of the concentrations tested affected Panx1 protein steady-state levels. (Figure 3 and Figure S4).

### 2.4. Effect of the Drug Panel on Panx1 mRNA Expression

**Figure 4:**
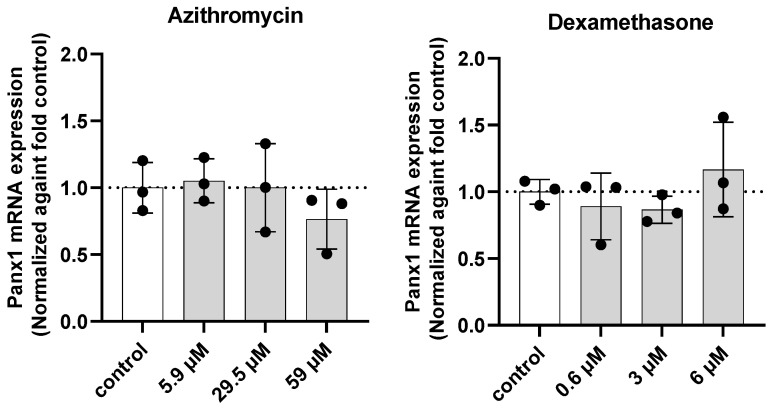

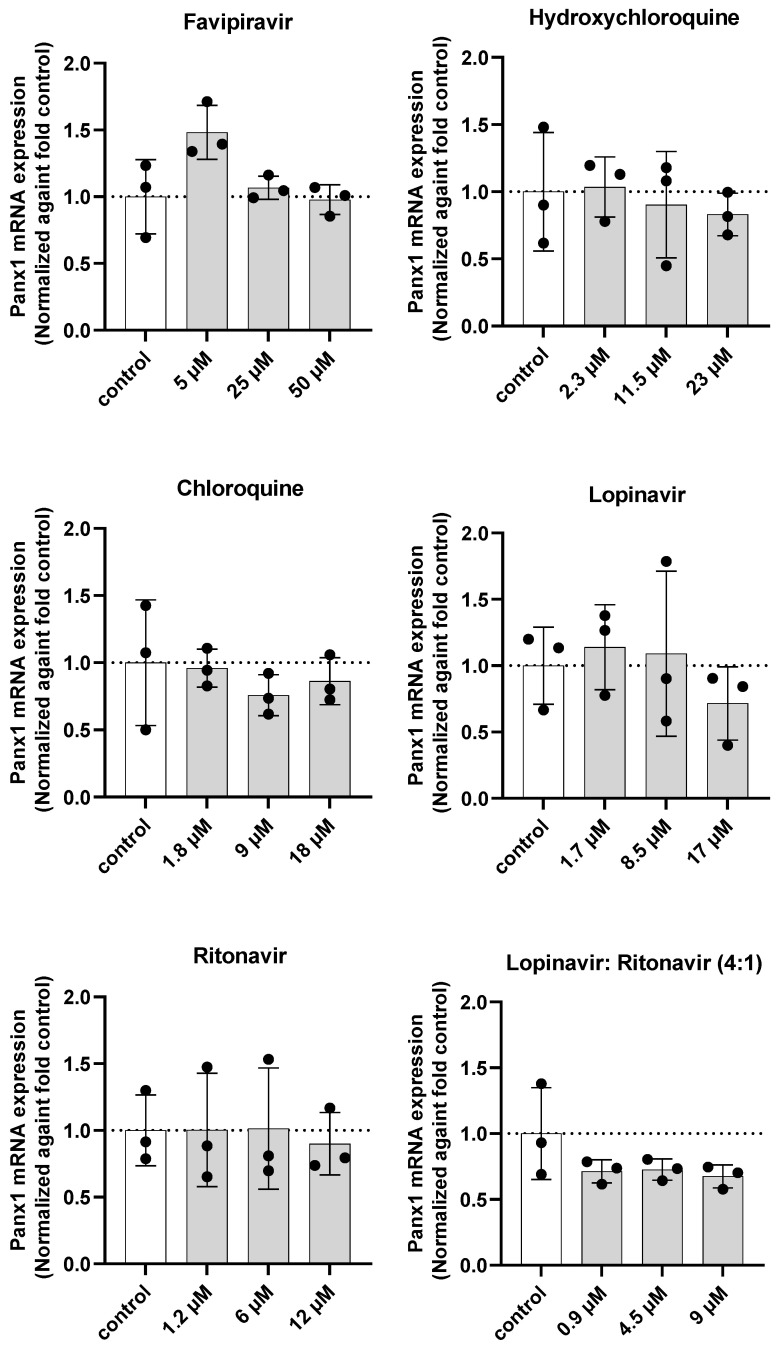

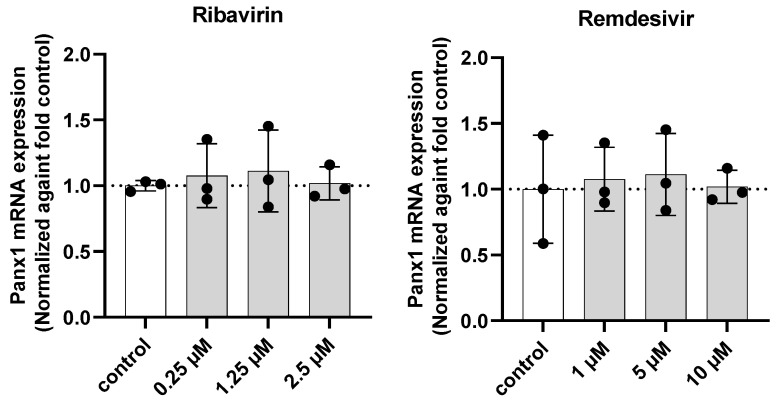
Panx1 mRNA expression after 24 h exposure of transduced Dubca cells overexpressing human Panx1 to the drug panel. mRNA expression levels were measured using RT-qPCR analysis. Data were analysed using the Pfaffl method in qbase+ and normalised using ubiquitin C as a housekeeping gene. Results were relatively expressed against the respective controls. Statistical analysis was performed with a parametric one-way ANOVA, followed by a Dunnett’s test. Dots represent the respective biological replicates (*N*). Data are expressed as mean ± standard deviation (*N* = 3, *n* = 3) and visualised in separate graphs per drug.

Drugs counteracting inflammation have been shown to downregulate Panx1 mRNA levels [58]. Despite the absence of effects at the translational level, Panx1 gene transcription could still be altered after 24 h exposure, as transcriptional changes might only translate at a later time point due to the relatively slow turnover rate of Panx1 proteins [62,63,65,66]. Transduced Dubca cells overexpressing human Panx1 were exposed for 24 h to the drug panel (Table 1), followed by RT-qPCR analysis. In line with the obtained results at the protein level (Figure 3), no effect of the drugs on Panx1 mRNA quantities was observed in any of the concentrations tested (Figure 4).

## 3. Discussion

The hypothesis of this study states that drugs formerly repurposed for the treatment of COVID-19 might act through alterations of Panx1 channels. Panx1 channels are key players in inflammation and were found to open upon SARS-CoV-2 infection, thus suggesting a potential role as drug targets [18,19]. The present study was set up to test the effects of drugs previously proposed for the treatment of COVID-19 on Panx1 channels at transcriptional, translational, and functional levels. As such, nine drugs and/or their combinations—namely, hydroxychloroquine, chloroquine, ribavirin, dexamethasone, azithromycin, remdesivir, favipiravir, lopinavir, ritonavir, and the combination of lopinavir and ritonavir—were included in this study. The majority of the drugs in the drug panel alter inflammatory processes in a direct or indirect way [23]. Some of the antiviral drugs, such as favipiravir, ribavirin, and the lopinavir: ritonavir combination, have been hypothesised to counteract inflammation simply by resolving the viral infection, while other drugs with antiviral effects, such as remdesivir, hydroxychloroquine, chloroquine, and azithromycin, are involved in the inhibition of NLRP3 inflammasome activation [23,33,67,68,69,70,71,72]. Dexamethasone, on the other hand, shows solely an anti-inflammatory effect but has been linked to alterations in inflammasome activity as well [73,74]. NLRP3 inflammasome activation plays an important role in the host immune response to SARS-CoV-2 infections, but its’ assembly and activation have also been frequently linked to Panx1 channel activity [12,16,75,76,77,78]. In addition, Panx1 channel inhibitors have proven to be able to inhibit the inflammasomes’ activation [78]. Hence, the NLRP3 inflammasome might connect the drugs’ potential activity in attenuating inflammation during SARS-CoV-2 infections and Panx1 channels. It should, however, be stressed that, with the exception of remdesivir and dexamethasone, the majority of these drugs have recently been found suboptimal or even non-efficient for repurposing as COVID-19 treatment by the European Medicines Agency (EMA) [79,80,81,82,83,84,85]. Nevertheless, the present study showed that hydroxychloroquine, favipiravir, lopinavir, and the combination of lopinavir and ritonavir inhibit Panx1 channel activity without affecting mRNA or protein levels after 24 h of exposure. Though current knowledge of the mechanisms underlying the activity of lopinavir and favipiravir cannot be traced back to NLRP3 inflammasome activation, these drugs can be linked to certain infectious diseases in which Panx1 channels are involved. Panx1 channels play critical roles in HIV infections, and lopinavir is a known HIV protease inhibitor [20,86]. Favipiravir is on the market as a viral RNA polymerase inhibitor and has been suggested as a drug candidate for the treatment of hepatitis C [21,87]. Hydroxychloroquine, on the other hand, was initially used as an antimalarial drug but has been repurposed for the treatment of inflammatory diseases, given its effect on cytokine production, as well as on NLRP3 inflammasome activation [88,89]. Hydroxychloroquine is directly connected with the NLRP3 inflammasome and was also proven to be the most potent inhibitor of Panx1-mediated ATP release in the present study. Due to severe side effects, this drug might not be an ideal candidate for repurposing for other Panx1-related diseases [90]. However, as a result of its implication in the treatment of COVID-19, interest in the drug was sparked, and a computational study identified new analogues with better safety profiles [91]. Seven new drugs and/or drug combinations have been authorised next to remdesivir for the use in COVID-19 patients by EMA—namely anakinra, regdanvimab, tocilizumab, sotrovimab, and the combinations of casirivimab and imdevimab, as well as the combination of PF-07321332 and ritonavir. These drugs, together with the new analogues of hydroxychloroquine, can form an interesting set of compounds for further investigations [92]. Due to similar links to inflammation, NLRP3 inflammasome activation, and Panx1-related infectious diseases, the proposed group of drugs could hold great potential to uncover additional Panx1 channel inhibitors [93,94,95,96,97,98]. To conclude, three new Panx1 channel inhibitors were identified from a panel of drugs formerly repurposed for the treatment of COVID-19 patients. This creates new opportunities for the treatment of Panx1-related diseases and also suggests that COVID-19 drug candidacy might be an interesting selection criterion in the search for new drugs targeting Panx1 channels.

## 4. Materials and Methods

### 4.1. Reagents and Chemicals

DMSO, dexamethasone (D4902), ribavirin (R9644), lopinavir (SML0491), ritonavir (SML1222), hydroxychloroquine sulphate (HO915), chloroquine diphosphate (C6628), and azithromycin dihydrate (A9834) were supplied by Sigma-Aldrich (Overijse, Belgium). In the case of the latter 3 drugs, the salt forms were purchased to avoid limitations due to the low solubility of the drugs. Remdesivir (30354-10) was purchased from Sanbio (Uden, The Netherlands), and favipiravir (FF29069) was obtained from Biosynth Carbosynth (Compton, United Kingdom). The peptide mimetic ^10^Panx1 was synthesised in-house by means of solid-phase peptide synthesis with a purity of >98%. Lanthanum trichloride was supplied by Merck Chemical n.v./s.a. (Overijse, Belgium). All other reagents were obtained from various suppliers at the highest analytical grade possible. Each drug was dissolved in the respective solvent (Table 1).

### 4.2. Cell Culture Setup and Maintenance

Transduced Dubca cells stably overexpressing human Panx1 (a 20-fold increase in Panx1 expression in comparison with the Dubca wild-type cell line) were thawed and seeded in a T75 flask (75 cm^2^) using Dulbecco’s modified Eagle medium (DMEM) containing Glutamax^TM^ (Gibco, Waltham, MA, USA), supplemented with 10% foetal bovine serum (FBS) (Gibco, Waltham, MA, USA), streptomycin, and penicillin (Thermo Fisher Scientific, Waltham, MA, USA). Cells were maintained at 37 °C in an incubator (5% CO_2_).

### 4.3. Cell Viability Assessment

Cell viability was assessed using a 3-(4,5-dimethylthiazol-2-yl)-2,5-diphenyltetrazolium bromide (MTT) (Sigma-Aldrich, Overijse, Belgium) assay. Transduced Dubca cells overexpressing human Panx1 were seeded in flat-bottom, 96-well culture plates (Corning, Glendale, AZ, USA) at a cell density of 12,000 cells/well (37,500 cells/cm^2^). Then, 24 h after seeding, cells were exposed to the drugs dissolved in 200 µL of phenol red-free DMEM (Gibco, Waltham, MA, USA), supplemented with Glutamax^TM^ (Gibco, Waltham, MA, USA), 10% FBS (Gibco, Waltham, MA, USA), streptomycin, and penicillin (Thermo Fisher Scientific, Waltham, MA, USA) for 24 h or in classic buffer (Tyrode buffer; 124 mM NaCl, 2.44 mM KCl, 10.82 mM NaHCO_3_, 0.38 mM NaHP0_4_ * H_2_O, 0.91 mM MgCl_2_ * 6 H_2_O, 1.82 mM CaCl_2_ * 6 H_2_O) for 45 min to a predetermined concentration range of each drug (Table 1). Cells were washed with 100 µL of warm phosphate-buffered saline (PBS) and incubated with MTT solution (0.5 mg/mL MTT in phenol red-free DMEM media) for 1.5 h. Next, each well was washed with 100 µL of PBS, and the water-insoluble formazan crystals were dissolved using 100 µL DMSO. After shaking for 10 min, absorbance was measured using a VICTOR3 Multilabel Plate Counter (PerkinElmer, Waltham, MA, USA) at a wavelength of 570 nm. Viability was expressed relative to the untreated control cells.

### 4.4. Panx1 Channel Activity Assay

Transduced Dubca cells overexpressing human Panx1 were seeded at a cell density of 12,000 cells per well (37,500 cells/cm^2^) in flat-bottom, 96-well culture plates (Corning, Glendale, AZ, USA) and incubated at 37 °C overnight (5% CO_2_). Drugs were dissolved in classic buffer (Tyrode buffer; 124 mM NaCl, 2.44 mM KCl, 10.82 mM NaHCO_3_, 0.38 mM NaHP0_4_ * H_2_O, 0.91 mM MgCl_2_ * 6 H_2_O, 1.82 mM CaCl_2_ * 6 H_2_O) and osmotic buffer (Tyrode buffer; 124 mM NaCl, 5 mM KCl, 10.82 mM NaHCO_3_, 0.38 mM NaHP0_4_ * H_2_O, 0.91 mM MgCl_2_ * 6 H_2_O, 1.82 mM CaCl_2_ * 6 H_2_O). Following a washout period of 30 min using a classic buffer, cells were preincubated with the drugs in a defined concentration range (Table 1) in a classic buffer for 15 min. Cells were subsequently exposed to the same range of concentrations of each drug (Table 1) in an osmotic buffer for 30 min to trigger the opening of Panx1 channels via osmotic shock. Throughout the procedure, cells and solutions were kept at 37 °C in an incubator (5% CO_2_). Next, 50 µL of each solution was transferred into white, opaque, 96-well plates (Corning, Glendale, AZ, USA), and extracellular ATP release was measured using an ATP Bioluminescent Assay Kit, following the manufacturer’s instructions (Sigma-Aldrich, Overijse, Belgium), in a VICTOR3 Multilabel Plate Counter (PerkinElmer, Waltham, MA, USA).

### 4.5. Immunoblot Analysis

Following 24 h of exposure to the drugs dissolved in DMEM culture medium (Table 1), transduced Dubca cells overexpressing human Panx1 were washed, scraped, and collected in the presence of ice-cold PBS. After centrifugation at 4332× *g* for 5 min at 4 °C, supernatants were removed, and pellets were resuspended in ice-cold PBS. Thereafter, cell suspensions were transferred to new tubes and subjected to an additional centrifugation step of 5 min at 2040× *g* at 4 °C. After removal of supernatants, the pellets were resuspended in lysis buffer (radio-immunoprecipitation assay buffer, 1% *v*/*v* ethylenediaminetetraacetic acid, and 1% *v*/*v* protease and phosphatase inhibitor cocktail; Thermo Fisher Scientific, Waltham, MA, USA) and sonicated for 30 s at 50% pulse. Subsequently, samples were shaken on a rotator for 15 min at 4 °C and centrifuged at 14,000× *g* for 15 min at 4 °C. Finally, supernatants were transferred to tubes and stored at −80 °C. Protein content was quantified via the Pierce^TM^ Bicinchoninic Acid Protein Assay Kit (Thermo Fisher Scientific, Waltham, MA, USA). Next, 20 µg of protein of each sample was loaded onto 10% Mini-PROTEAN^®^ TGX Stain-Free™ precast gels (Bio-Rad, Hercules, CA, USA). After electrophoreses and blotting, nitrocellulose membranes (Bio-Rad, Hercules, CA, USA) were blocked using a blocking buffer containing 5% *w*/*v* non-fat milk (Régilait, Saint-Martin-Belle-Roche, France) in a Tris-buffered saline solution (20 mM Tris and 135 mM sodium chloride) containing 0.1% *v*/*v* Tween-20 (Sigma-Aldrich, Overijse, Belgium). Membranes were incubated overnight at 4 °C, with primary antibodies directed against Panx1 (Panx1 rabbit mAb; Bioké, Leiden, The Netherlands) diluted in blocking buffer (1:1000), followed by an additional incubation for 1 h at room temperature with polyclonal goat anti-rabbit secondary antibody (1:1000) (Dako, Santa Clara, CA, USA). Proteins were detected using enhanced chemiluminescence, and Image Lab 6.1 software was used for densiometric analysis. For semi-quantification purposes, a normalisation method based on total protein loading was used to overcome the drawbacks associated with housekeeping proteins [59]. Panx1 signals were normalised against total protein and expressed relatively to Panx1 protein levels of untreated Dubca cells overexpressing Panx1.

### 4.6. Real-Time Quantitative Reverse-Transcription Polymerase Chain Reaction Analysis

Cell pellets for RNA extraction were collected following exposure of transduced Dubca cells overexpressing human Panx1 to the drug panel for 24 h (Table 1). Pellets were stored at −80 °C. Total RNA was extracted using a GenElute^TM^ Mammalian Total RNA purification Miniprep Kit (Sigma-Aldrich, Overijse, Belgium) and the On-column DNase I digestion Set (Sigma-Aldrich, Overijse, Belgium) according to the manufacturer’s instructions. Isolated RNA was spectrophotometrically measured using a NanoDrop^®^ 2000 Spectrophotometer (Thermo Fisher Scientific, Waltham, MA, USA) to assess RNA yield and purity. A cut-off ratio between 1.8 and 2.1 for the absorption at 260/280 nm was set. Synthesis and amplification of cDNA were performed via an iScript^TM^ cDNA Synthesis Kit (Bio-Rad, Hercules, CA, USA), using a MiniAmp Plus Thermal Cycler (Thermo Fisher Scientific, Waltham, MA, USA). Samples were purified using a GenElute™ PCR Clean-Up Kit (Sigma-Aldrich, Overijse, Belgium) according to the manufacturer’s protocol. Samples were tested on an Applied Biosystems QuantStudio 3 Real-Time PCR system (Thermo Fisher Scientific, Waltham, MA, USA) using TaqMan^®^ Gene Expression Assays (Applied Biosystems, Waltham, MA, USA). TaqMan probes and primers specific for the target and reference gene used for RT-qPCR analysis are depicted in Table 2. Relative alterations in mRNA levels were calculated in comparison with the untreated control according to the Pfaffl method [99]. Primer efficiencies lie within the range of 90% to 110%.

### 4.7. Statistical Analysis

All experiments were performed in 3 different passages of transduced Dubca cells overexpressing human Panx1 (*N* = 3). The number of technical replicates (n) is specified in the figure legends. Data are presented as means ± standard deviation. The normal distribution of the data sets was assessed by means of a Shapiro–Wilk test. Depending on the degree of normality, results were analysed with a parametric one-way analysis of variance (ANOVA), followed by a Dunnett’s post hoc test or non-parametric Kruskal–Wallis test, followed by a Dunn’s multiple comparisons test. Significance levels are indicated according to the following symbols: * *p* ≤ 0.05 ** *p* ≤ 0.01 *** *p* ≤ 0.001 and **** *p* ≤ 0.0001. Statistical analysis was performed in GraphPad^®^ Prism 9 software (GraphPad^®^ Software Inc., San Diego, CA, USA).

## Figures and Tables

**Table 1:** Panel of drugs and drug combinations tested in the present study: DMSO, dimethyl sulfoxide; RT-qPCR, real-time quantitative reverse-transcription polymerase chain reaction analysis; C_max_, maximum plasma concentration extracted from literature and recalculated to values in µM. References are cited between square brackets behind their respective C_max_ value; CC_10_, concentration inducing 10% of cell death).

Drug	Solvent	C_max_ (µM)	CC_10_ (µM)	Final Concentration Range Tested for CC_10_ Determination (µM)	Logarithmic Value of the Final Concentration Range Tested for CC_10_ Determination (µM)	Concentration Range Tested for Functional Analysis (µM)	Concentration Range Tested for Expression Analysis (µM)
Azithromycin (dihydrate)	DMSO	0.52 [24]	59	1–10–25–50–100–200	0–1–1.4–1.7–2–2.3	5.9–29.5–59–118–295–590	5.9–29.5–59
Ritonavir	DMSO	22.5 [25]	12	1–10–25–50–100–200	0–1–1.4–1.7–2–2.3	1.2–6–12–24–60–120	1.2–6–12
Lopinavir	DMSO	19.0 [26]	17	1–10–25–50–100–200	0–1–1.4–1.7–2–2.3	1.7–8.5–17–34–85–170	1.7–8.5–17
Lopinavir: Ritonavir (4:1)	DMSO	19.0 [26]	9	1–10–25–50–100–200	0–1–1.4–1.7–2–2.3	0.9–4.5–9–18–45–90	0.9–4.5–9
Remdesivir	water	9.03 [27]	>100	0.01–0.1–1–10–50–100	−2–−1–0–1–1.7–2	1–5–10–20–50–100	1–5–10
Dexamethasone	DMSO	0.63 [28]	>200	0.1–0.5–1–10–50–100–200	−1–−0.30–0–1–1.7–2–2.3	0.6–3–6–12–30–60	0.6–3–6
Favipiravir	water	53.4 [29]	>200	1–10–25–50–100–200	0–1–1.4–1.7–2–2.3	5–25–50–100–250–500	5–25–50
Hydroxychloroquine (sulphate)	water	0.97 [30]	23	1–10–25–50–100–200	0–1–1.4–1.7–2–2.3	2.3–11.5–23–46–115–230	2.3–11.5–23
Chloroquine (diphosphate)	water	0.81 [31]	18	1–10–25–50–100–200	0–1–1.4–1.7–2–2.3	1.8–9–18–36–90–180	1.8–9–18
Ribavirin	water	2.63 [32]	2.5	1–10–25–50–100–200–400	0–1–1.4–1.7–2–2.3–2.6	0.25–1.25–2.5–5–12.5–25	0.25–1.25–2.5

**Table 2:** Primers and probes for RT-qPCR analysis. (Panx1 (pannexin1); UBC (ubiquitin C)).

Gene Symbol	Assay ID	Accession Number	Assay Location	Amplicon Size	Accession Number
UBC	Hs01871556-s1	M26880.1	2173	135	-
Panx1	Hs00209791-m1	NM_015368.3	929	90	3–4
		XM_011542734.2	539		4–5
		XM_017017464.1	874		4–5

## Data Availability

Data are available upon request.

## Supplementary Materials

The following supporting information can be downloaded at: https://www.mdpi.com/article/10.3390/ijms23105664/s1.

## References

[1] COVID-19 Map-Johns Hopkins Coronavirus Resource Center. https://coronavirus.jhu.edu/map.html.

[2] Zhang B., Zhou X., Qiu Y., Song Y., Feng F., Feng J., Song Q., Jia Q., Wang J. (2020). Clinical Characteristics of 82 Cases of Death from COVID-19. PLoS ONE.

[3] Chen G., Wu D., Guo W., Cao Y., Huang D., Wang H., Wan T., Zhang X., Chen H., Yu H. (2020). Clinical and Immunological Features of Severe and Moderate Coronavirus Disease 2019. J. Clin. Investig..

[4] Li C., He Q., Qian H., Liu J. (2021). Overview of the Pathogenesis of COVID-19. Exp. Ther. Med..

[5] Crespo Yanguas S., Willebrords J., Johnstone S.R., Maes M., Decrock E., De Bock M., Leybaert L., Cogliati B., Vinken M. (2017). Pannexin1 as Mediator of Inflammation and Cell Death. Biochim. Biophys. Acta.

[6] Koval M., Cwiek A., Carr T., Good M.E., Lohman A.W., Isakson B.E. (2021). Pannexin 1 as a Driver of Inflammation and Ischemia–Reperfusion Injury. Purinergic Signal..

[7] Vasseur M.L., Lelowski J., Bechberger J.F., Sin W.C., Naus C.C. (2014). Pannexin 2 Protein Expression Is Not Restricted to the CNS. Front. Cell. Neurosci..

[8] Bruzzone R., Hormuzdi S.G., Barbe M.T., Herb A., Monyer H. (2003). Pannexins, a Family of Gap Junction Proteins Expressed in Brain. Proc. Natl. Acad. Sci. USA.

[9] Medina C.B., Mehrotra P., Arandjelovic S., Perry J.S.A., Guo Y., Morioka S., Barron B., Walk S.F., Ghesquière B., Krupnick A.S. (2020). Metabolites Released from Apoptotic Cells Act as Tissue Messengers. Nature.

[10] Narahari A.K., Kreutzberger A.J.B., Gaete P.S., Chiu Y.H., Leonhardt S.A., Medina C.B., Jin X., Oleniacz P.W., Kiessling V., Barrett P.Q. (2021). Atp and Large Signaling Metabolites Flux through Caspase-Activated Pannexin 1 Channels. Elife.

[11] Chekeni F.B., Elliott M.R., Sandilos J.K., Walk S.F., Kinchen J.M., Lazarowski E.R., Armstrong A.J., Penuela S., Laird D.W., Salvesen G.S. (2010). Pannexin 1 Channels Mediate ‘Find-Me’ Signal Release and Membrane Permeability during Apoptosis. Nature.

[12] Chen K.W., Demarco B., Broz P. (2020). Pannexin-1 Promotes NLRP3 Activation during Apoptosis but Is Dispensable for Canonical or Noncanonical Inflammasome Activation. Eur. J. Immunol..

[13] Makarenkova H.P., Shah S.B., Shestopalov V.I. (2018). The Two Faces of Pannexins: New Roles in Inflammation and Repair. J. Inflamm. Res..

[14] Sanchez Arias J.C., Wicki-Stordeur L.E., Candlish R.C., van der Slagt E., Paci I., Rao P.P.N., MacVicar B.A., Swayne L.A. (2020). PANX1 in Inflammation Heats up: New Mechanistic Insights with Implications for Injury and Infection. Cell Calcium.

[15] Yin F., Zheng P.q., Zhao L.q., Wang Y.z., Miao N.j., Zhou Z.l., Cheng Q., Chen P.p., Xie H.y., Li J.y. (2021). Caspase-11 Promotes NLRP3 Inflammasome Activation via the Cleavage of Pannexin1 in Acute Kidney Disease. Acta Pharmacol. Sin..

[16] Demarco B., Chen K.W., Broz P. (2019). Pannexin-1 Channels Bridge Apoptosis to NLRP3 Inflammasome Activation. Mol. Cell. Oncol..

[17] Lohman A.W., Leskov I.L., Butcher J.T., Johnstone S.R., Stokes T.A., Begandt D., Delalio L.J., Best A.K., Penuela S., Leitinger N. (2015). Pannexin 1 Channels Regulate Leukocyte Emigration through the Venous Endothelium during Acute Inflammation. Nat. Commun..

[18] Luu R., Valdebenito S., Scemes E., Cramer-Bordé E., Bomsel M., Eugenin E. (2021). Pannexin-1 Channel Opening Is Critical for COVID-19 Pathogenesis. ISCIENCE.

[19] Swayne L.A., Johnstone S.R., Ng C.S., Sanchez-Arias J.C., Good M.E., Penuela S., Lohman A.W., Wolpe A.G., Laubach V.E., Michael Koval X. (2020). The Pathophysiology of COVID-19 and SARS-CoV-2 Infection: Consideration of Pannexin 1 Channels in COVID-19 Pathology and Treatment. Am. J. Physiol.-Lung Cell. Mol. Physiol..

[20] Orellana J.A., Velasquez S., Williams D.W., Sáez J.C., Berman J.W., Eugenin E.A. (2013). Pannexin1 Hemichannels Are Critical for HIV Infection of Human Primary CD4+ T Lymphocytes. J. Leukoc. Biol..

[21] Kim O.K., Nam D.E., Hahn Y.S. (2021). The Pannexin 1/Purinergic Receptor P2X4 Pathway Controls the Secretion of MicroRNA-Containing Exosomes by HCV-Infected Hepatocytes. Hepatology.

[22] COVID-19 Studies from the World Health Organization Database—ClinicalTrials.Gov. https://clinicaltrials.gov/ct2/who_table.

[23] Gilzad-Kohan H., Jamali F. (2020). View of Anti-Inflammatory Properties of Drugs Used to Control COVID-19 and Their Effects on the Renin-Angiotensin System and Angiotensin-Converting Enzyme-2. J. Pharm. Pharm. Sci..

[24] FDA ZITHROMAX^®^ (Azithromycin Tablets) and (Azithromycin for Oral Suspension). https://www.accessdata.fda.gov/drugsatfda_docs/label/2013/050710s039,050711s036,050784s023lbl.pdf.

[25] Gatti G., Di Biagio A., Casazza R., De Pascalis C., Bassetti M., Cruciani M., Vella S.B.D. (1999). The Relationship between Ritonavir Plasma Levels and Side-Effects: Implications for Therapeutic Drug Monitoring. AIDS Res. Treat..

[26] Jackson A., Hill A., Puls R., Else L., Amin J., Back D., Lin E., Khoo S., Emery S., Morley R. (2011). Pharmacokinetics of Plasma Lopinavir/Ritonavir Following the Administration of 400/100 Mg, 200/150 Mg and 200/50 Mg Twice Daily in HIV-Negative Volunteers. J. Antimicrob. Chemother..

[27] EMA Summary on Compassionate Use Remdesivir Gilead. https://www.ema.europa.eu/en/documents/other/summary-compassionate-use-remdesivir-gilead_en.pdf.

[28] FDA Clinical Pharmacology and Biopharmaceutics Review(s) Application Number: 211379 Orig1s000. https://www.accessdata.fda.gov/drugsatfda_docs/nda/2019/211379Orig1s000ClinPharmR.pdf.

[29] Saglam O., Demiray G., Güney B., Dogan-Kurtoglu E., Ulusoy M.G., Saraner N., Sevici G., Nacak M., Erenmemisoglu A., Tüzer V. (2020). Single Dose, Two-Way Crossover Bioequivalence Study of Favipiravir Tablet in Healthy Male Subjects. J. Pharm. Drug Dev..

[30] Tett S., Day R., Cutler D. (1996). Hydroxychloroquine Relative Bioavailability: Within Subject Reproducibility. Br. J. Clin. Pharmacol..

[31] Cui C., Zhang M., Yao X., Tu S., Hou Z., Jie En V.S., Xiang X., Lin J., Cai T., Shen N. (2020). Dose Selection of Chloroquine Phosphate for Treatment of COVID-19 Based on a Physiologically Based Pharmacokinetic Model. Acta Pharm. Sin. B.

[32] Glue P., Schenker S., Gupta S., Clement R.P., Zambas D., Salfi M. (2000). The Single Dose Pharmacokinetics of Ribavirin in Subjects with Chronic Liver Disease. Br. J. Clin. Pharmacol..

[33] Foley K., Kast R.E., Altschuler E.L. (2009). Ritonavir and Disulfiram Have Potential to Inhibit Caspase-1 Mediated Inflammation and Reduce Neurological Sequelae after Minor Blast Exposure. Med. Hypotheses.

[34] Cao B., Wang Y., Wen D., Liu W., Wang J., Fan G., Ruan L., Song B., Cai Y., Wei M. (2020). A Trial of Lopinavir–Ritonavir in Adults Hospitalized with Severe Covid-19. N. Engl. J. Med..

[35] Zeldin R.K., Petruschke R.A. (2004). Pharmacological and Therapeutic Properties of Ritonavir-Boosted Protease Inhibitor Therapy in HIV-Infected Patients. J. Antimicrob. Chemother..

[36] Nosita F., Pirzada K., Lestari T., Cahyono R. (2020). Should Azithromycin Be Used to Treat COVID-19? A Rapid Review. BJGP Open.

[37] Damle B., Vourvahis M., Wang E., Leaney J., Corrigan B. (2020). Clinical Pharmacology Perspectives on the Antiviral Activity of Azithromycin and Use in COVID-19. Clin. Pharmacol. Ther..

[38] Yang J., Guo Z., Liu X., Liu Q., Wu M., Yao X., Liu Y., Cui C., Li H., Song C. (2020). Cytotoxicity Evaluation of Chloroquine and Hydroxychloroquine in Multiple Cell Lines and Tissues by Dynamic Imaging System and Physiologically Based Pharmacokinetic Model. Front. Pharmacol..

[39] Wang M., Cao R., Zhang L., Yang X., Liu J., Xu M., Shi Z., Hu Z., Zhong W., Xiao G. (2020). Remdesivir and Chloroquine Effectively Inhibit the Recently Emerged Novel Coronavirus (2019-NCoV) in Vitro. Cell Res..

[40] Sheahan T.P., Sims A.C., Leist S.R., Schäfer A., Won J., Brown A.J., Montgomery S.A., Hogg A., Babusis D., Clarke M.O. (2020). Comparative Therapeutic Efficacy of Remdesivir and Combination Lopinavir, Ritonavir, and Interferon Beta against MERS-CoV. Nat. Commun..

[41] Vinken M., Hengstler J.G. (2018). Characterization of Hepatocyte-Based in Vitro Systems for Reliable Toxicity Testing. Arch. Toxicol..

[42] Maes M., Vanhaecke T., Cogliati B., Yanguas S.C., Willebrords J., Rogiers V., Vinken M. (2015). Measurement of Apoptotic and Necrotic Cell Death in Primary Hepatocyte Cultures. Methods Mol. Biol..

[43] Elliott M.R., Chekeni F.B., Trampont P.C., Lazarowski E.R., Kadl A., Walk S.F., Park D., Woodson R.I., Ostankovich M., Sharma P. (2009). Nucleotides Released by Apoptotic Cells Act as a Find-Me Signal for Phagocytic Clearance. Nature.

[44] Hattori M., Gouaux E. (2012). Molecular Mechanism of ATP Binding and Ion Channel Activation in P2X Receptors. Nature.

[45] Wilkaniec A., Gąssowska M., Czapski G.A., Cieślik M., Sulkowski G., Adamczyk A. (2017). P2X7 Receptor-Pannexin 1 Interaction Mediates Extracellular Alpha-Synuclein-Induced ATP Release in Neuroblastoma SH-SY5Y Cells. Purinergic Signal..

[46] Karmakar M., Katsnelson M.A., Dubyak G.R., Pearlman E. (2016). Neutrophil P2X7 Receptors Mediate NLRP3 Inflammasome-Dependent IL-1β Secretion in Response to ATP. Nat. Commun..

[47] Pelegrin P. (2021). P2X7 Receptor and the NLRP3 Inflammasome: Partners in Crime. Biochem. Pharmacol..

[48] Paoletti A., Raza S.Q., Voisin L., Law F., Caillet M., Martins I., Deutsch E., Perfettini J.-L. (2013). Pannexin-1-the Hidden Gatekeeper for HIV-1. J. Leukoc. Biol..

[49] Gorska A.M., Donoso M., Valdebenito S., Prideaux B., Queen S., Scemes E., Clements J., Eugenin E. (2021). Human Immunodeficiency Virus-1/Simian Immunodeficiency Virus Infection Induces Opening of Pannexin-1 Channels Resulting in Neuronal Synaptic Compromise: A Novel Therapeutic Opportunity to Prevent NeuroHIV. J. Neurochem..

[50] Michalski K., Kawate T. (2016). Carbenoxolone Inhibits Pannexin1 Channels through Interactions in the First Extracellular Loop. J. Gen. Physiol..

[51] Ma W., Hui H., Pelegrin P., Surprenant A. (2009). Pharmacological Characterization of Pannexin-1 Currents Expressed in Mammalian Cells. J. Pharmacol. Exp. Ther..

[52] Feig J.L., Mediero A., Corciulo C., Liu H., Zhang J., Perez-Aso M., Picard L., Wilder T., Cronstein B. (2017). The Antiviral Drug Tenofovir, an Inhibitor of Pannexin-1-Mediated ATP Release, Prevents Liver and Skin Fibrosis by Downregulating Adenosine Levels in the Liver and Skin. PLoS ONE.

[53] Orellana J.A., Shoji K.F., Abudara V., Ezan P., Amigou E., Sáez P.J., Jiang J.X., Naus C.C., Sáez J.C., Giaume C. (2011). Amyloid β-Induced Death in Neurons Involves Glial and Neuronal Hemichannels. J. Neurosci..

[54] Pelegrin P., Barroso-Gutierrez C., Surprenant A. (2008). P2X7 Receptor Differentially Couples to Distinct Release Pathways for IL-1beta in Mouse Macrophage. J. Immunol..

[55] Pelegrin P., Surprenant A. (2006). Pannexin-1 Mediates Large Pore Formation and Interleukin-1β Release by the ATP-Gated P2X7 Receptor. EMBO J..

[56] Basova L.V., Tang X., Umasume T., Gromova A., Zyrianova T., Shmushkovich T., Wolfson A., Hawley D., Zoukhri D., Shestopalov V.I. (2017). Manipulation of Panx1 Activity Increases the Engraftment of Transplanted Lacrimal Gland Epithelial Progenitor Cells. Investig. Ophthalmol. Vis. Sci..

[57] Yang Y., Delalio L.J., Best A.K., Macal E., Milstein J., Donnelly I., Miller A.M., McBride M., Shu X., Koval M. (2020). Endothelial Pannexin 1 Channels Control Inflammation by Regulating Intracellular Calcium. J. Immunol..

[58] Huang G., Bao J., Shao X., Zhou W., Wu B., Ni Z., Wang L. (2020). Inhibiting Pannexin-1 Alleviates Sepsis-Induced Acute Kidney Injury via Decreasing NLRP3 Inflammasome Activation and Cell Apoptosis. Life Sci..

[59] Eaton S.L., Roche S.L., Llavero Hurtado M., Oldknow K.J., Farquharson C., Gillingwater T.H., Wishart T.M. (2013). Total Protein Analysis as a Reliable Loading Control for Quantitative Fluorescent Western Blotting. PLoS ONE.

[60] Boassa D., Qiu F., Dahl G., Sosinsky G. (2009). Cell Communication & Adhesion Trafficking Dynamics of Glycosylated Pannexin1 Proteins Trafficking Dynamics of Glycosylated Pannexin1 Proteins. Cell Commun. Adhes..

[61] Penuela S., Bhalla R., Nag K., Laird D.W. (2009). Glycosylation Regulates Pannexin Intermixing and Cellular Localization. Mol. Biol. Cell.

[62] Boassa D., Ambrosi C., Qiu F., Dahl G., Gaietta G., Sosinsky G. (2007). Pannexin1 Channels Contain a Glycosylation Site That Targets the Hexamer to the Plasma Membrane. J. Biol. Chem..

[63] Gehi R., Shao Q., Laird D.W. (2011). Pathways Regulating the Trafficking and Turnover of Pannexin1 Protein and the Role of the C-Terminal Domain. J. Biol. Chem..

[64] Penuela S., Simek J., Thompson R.J. (2014). Regulation of Pannexin Channels by Post-Translational Modifications. FEBS Lett..

[65] Penuela S., Bhalla R., Gong X.Q., Cowan K.N., Celetti S.J., Cowan B.J., Bai D., Shao Q., Laird D.W. (2007). Pannexin 1 and Pannexin 3 Are Glycoproteins That Exhibit Many Distinct Characteristics from the Connexin Family of Gap Junction Proteins. J. Cell Sci..

[66] Liu Y., Beyer A., Aebersold R. (2016). On the Dependency of Cellular Protein Levels on MRNA Abundance. Cell.

[67] Fujita Y., Matsuoka N., Temmoku J., Furuya M.Y., Asano T., Sato S., Kobayashi H., Watanabe H., Suzuki E., Urano T. (2019). Hydroxychloroquine Inhibits IL-1β Production from Amyloid-Stimulated Human Neutrophils. Arthritis Res. Ther..

[68] Fan L.C., Lin J.L., Yang J.W., Mao B., Lu H.W., Ge B.X., Choi A.M.K., Xu J.F. (2017). Macrolides Protect against Pseudomonas Aeruginosa Infection via Inhibition of Inflammasomes. Am. J. Physiol.-Lung Cell. Mol. Physiol..

[69] Chen X., Wang N., Zhu Y., Lu Y., Liu X., Zheng J. (2017). The Antimalarial Chloroquine Suppresses LPS-Induced NLRP3 Inflammasome Activation and Confers Protection against Murine Endotoxic Shock. Mediators Inflamm..

[70] Lendermon E.A., Coon T.A., Bednash J.S., Weathington N.M., McDyer J.F., Mallampalli R.K. (2017). Azithromycin Decreases NALP3 MRNA Stability in Monocytes to Limit Inflammasome-Dependent Inflammation. Respir. Res..

[71] Yin L., Zhao H., Zhang H., Li Y., Dong Y., Ju H., Kong F., Zhao S. (2021). Remdesivir Alleviates Acute Kidney Injury by Inhibiting the Activation of NLRP3 Inflammasome. Front. Immunol..

[72] Bahadoram M., Keikhaei B., Saeedi-Boroujeni A., Mahmoudian-Sani M.R. (2021). Chloroquine/Hydroxychloroquine: An Inflammasome Inhibitor in Severe COVID-19?. Naunyn. Schmiedebergs. Arch. Pharmacol..

[73] Guan M., Ma H., Fan X., Chen X., Miao M., Wu H. (2020). Dexamethasone Alleviate Allergic Airway Inflammation in Mice by Inhibiting the Activation of NLRP3 Inflammasome. Int. Immunopharmacol..

[74] Yang J.W., Mao B., Tao R.J., Fan L.C., Lu H.W., Ge B.X., Xu J.F. (2020). Corticosteroids Alleviate Lipopolysaccharide-Induced Inflammation and Lung Injury via Inhibiting NLRP3-Inflammasome Activation. J. Cell. Mol. Med..

[75] Zeng J., Xie X., Feng X.-L., Xu L., Han J.-B., Yu D., Zou Q.-C., Liu Q., Li X., Ma G. (2022). Specific Inhibition of the NLRP3 Inflammasome Suppresses Immune Overactivation and Alleviates COVID-19 like Pathology in Mice. eBioMedicine.

[76] Zhao N., Di B., Xu L.L. (2021). The NLRP3 Inflammasome and COVID-19: Activation, Pathogenesis and Therapeutic Strategies. Cytokine Growth Factor Rev..

[77] Pan P., Shen M., Yu Z., Ge W., Chen K., Tian M., Xiao F., Wang Z., Wang J., Jia Y. (2021). SARS-CoV-2 N Protein Promotes NLRP3 Inflammasome Activation to Induce Hyperinflammation. Nat. Commun..

[78] Silverman W.R., de Rivero Vaccari J.P., Locovei S., Qiu F., Carlsson S.K., Scemes E., Keane R.W., Dahl G. (2009). The Pannexin 1 Channel Activates the Inflammasome in Neurons and Astrocytes. J. Biol. Chem..

[79] Hinks T.S.C., Cureton L., Knight R., Wang A., Cane J.L., Barber V.S., Black J., Dutton S.J., Melhorn J., Jabeen M. (2021). Azithromycin versus Standard Care in Patients with Mild-to-Moderate COVID-19 (ATOMIC2): An Open-Label, Randomised Trial. Lancet Respir. Med..

[80] Tortajada C., Colomer E., Andreu-Ballester J.C., Esparcia A., Oltra C., Flores J. (2021). Corticosteroids for COVID-19 Patients Requiring Oxygen Support? Yes, but Not for Everyone: Effect of Corticosteroids on Mortality and Intensive Care Unit Admission in Patients with COVID-19 According to Patients’ Oxygen Requirements. J. Med. Virol..

[81] Patel T.K., Patel P.B., Barvaliya M., Saurabh M.K., Bhalla H.L., Khosla P.P. (2021). Efficacy and Safety of Lopinavir-Ritonavir in COVID-19: A Systematic Review of Randomized Controlled Trials. J. Infect. Public Health.

[82] Hassanipour S., Arab-Zozani M., Amani B., Heidarzad F., Fathalipour M., Martinez-de-Hoyo R. (2021). The Efficacy and Safety of Favipiravir in Treatment of COVID-19: A Systematic Review and Meta-Analysis of Clinical Trials. Sci. Rep..

[83] Gottlieb R.L., Vaca C.E., Paredes R., Mera J., Webb B.J., Perez G., Oguchi G., Ryan P., Nielsen B.U., Brown M. (2022). Early Remdesivir to Prevent Progression to Severe Covid-19 in Outpatients. N. Engl. J. Med..

[84] Aljadeed R. (2021). The Rise and Fall of Hydroxychloroquine and Chloroquine in COVID-19. J. Pharm. Pract..

[85] Tong S., Su Y., Yu Y., Wu C., Chen J., Wang S., Jiang J. (2020). Ribavirin Therapy for Severe COVID-19: A Retrospective Cohort Study. Int. J. Antimicrob. Agents.

[86] Barragan P., Podzamczer D. (2008). Lopinavir/Ritonavir: A Protease Inhibitor for HIV-1 Treatment. Expert Opin. Pharmacother..

[87] De Ávila A.I., Gallego I., Soria M.E., Gregori J., Quer J., Ignacio Esteban J., Rice C.M., Domingo E., Perales C. (2016). Lethal Mutagenesis of Hepatitis C Virus Induced by Favipiravir. PLoS ONE.

[88] Schrezenmeier E., Dörner T. (2020). Mechanisms of Action of Hydroxychloroquine and Chloroquine: Implications for Rheumatology. Nat. Rev. Rheumatol..

[89] Tang T.T., Lv L.L., Pan M.M., Wen Y., Wang B., Li Z.L., Wu M., Wang F.M., Crowley S.D., Liu B.C. (2018). Hydroxychloroquine Attenuates Renal Ischemia/Reperfusion Injury by Inhibiting Cathepsin Mediated NLRP3 Inflammasome Activation. Cell Death Dis..

[90] McChesney E.W. (1983). Animal Toxicity and Pharmacokinetics of Hydroxychloroquine Sulfate. Am. J. Med..

[91] Sinha M., Gupta A., Gupta S., Singh P., Pandit S., Chauhan S.S., Parthasarathi R. (2021). Analogue Discovery of Safer Alternatives to HCQ and CQ Drugs for SAR-CoV-2 by Computational Design. Comput. Biol. Med..

[92] COVID-19 Treatments|European Medicines Agency. https://www.ema.europa.eu/en/human-regulatory/overview/public-health-threats/coronavirus-disease-covid-19/treatments-vaccines/covid-19-treatments.

[93] Diamond M.S., Kanneganti T.-D. (2022). Innate Immunity: The First Line of Defense against SARS-CoV-2. Nat. Immunol..

[94] Collotta D., Hull W., Mastrocola R., Chiazza F., Cento A.S., Murphy C., Verta R., Alves G.F., Gaudioso G., Fava F. (2020). Baricitinib Counteracts Metaflammation, Thus Protecting against Diet-Induced Metabolic Abnormalities in Mice. Mol. Metab..

[95] Purvis G.S.D., Collino M., Aranda-Tavio H., Chiazza F., O’Riordan C.E., Zeboudj L., Mohammad S., Collotta D., Verta R., Guisot N.E.S. (2020). Inhibition of Bruton’s TK Regulates Macrophage NF-ΚB and NLRP3 Inflammasome Activation in Metabolic Inflammation. Br. J. Pharmacol..

[96] Toribio M., Burdo T.H., Fulda E.S., Cetlin M., Chu S.M., Feldpausch M.N., Robbins G.K., Neilan T.G., Melbourne K., Grinspoon S.K. (2020). Effects of Integrase Inhibitor–Based ART on the NLRP3 Inflammasome Among ART-Naïve People With HIV. Open Forum Infect. Dis..

[97] Freeman T.L., Swartz T.H. (2020). Targeting the NLRP3 Inflammasome in Severe COVID-19. Front. Immunol..

[98] Iannitti R.G., Napolioni V., Oikonomou V., De Luca A., Galosi C., Pariano M., Massi-Benedetti C., Borghi M., Puccetti M., Lucidi V. (2016). IL-1 Receptor Antagonist Ameliorates Inflammasome-Dependent Inflammation in Murine and Human Cystic Fibrosis. Nat. Commun..

[99] Pfaffl M.W. (2001). A New Mathematical Model for Relative Quantification in Real-Time RT-PCR. Nucleic Acids Res..

