# Supplementary material for: Effects of Drugs Formerly Suggested for COVID-19 Repurposing on Pannexin1 Channels
Source: Int J Mol Sci. 2022 May 18;23(10):5664. doi: 10.3390/ijms23105664 (PMC9146942; doi:10.3390/ijms23105664)
Supplement: Supplementary file 1 [file ijms-23-05664-s001.zip › ijms-1664211-supplementaryy.pdf]

Effects of Drugs Formerly Suggested for COVID-19 Repurposing on Pannexin1 Channels

Anne Caufriez [1,2], Andrés Tabernilla [1], Raf Van Campenhout [1], Axelle Cooreman [1], Kaat Leroy [1], Julen Sanz Serrano[1], Prashant Kadam[1], Bruna dos Santos Rodrigues [1], Arthur Lamouroux [2], Steven Ballet [2,] †, Mathieu Vinken [1, †,*]

[1] Department of Pharmaceutical and Pharmacological Sciences, Vrije Universiteit Brussel, Laarbeeklaan 103, 1090 Brussels, Belgium; anne.caufriez@vub.be (A.C.); andres.tabernilla.garcia@vub.be (A.T.); raf.van.campenhout@vub.be (R.V.C.); axelle.cooreman@vub.be (A.C.); kaat.leroy@vub.be (K.L.); julen.sanz.serrano@vub.be (J.S.S.); prashant.kadam@vub.be (P.K.); bruna.dos.santos.rodrigues@vub.be (B.d.S.R.)

[2] Departments of Chemistry and Bioengineering Sciences, Vrije Universiteit Brussel, Pleinlaan 2, 1050 Brussels, Belgium; anne.caufriez@vub.be (A.C.); arthur.lamouroux@vub.be (A.L.); steven.ballet@vub.be (S.B.)

* Correspondence: mathieu.vinken@vub.be; Tel.: +32-2477-4587

† These authors contributed equally to this work.

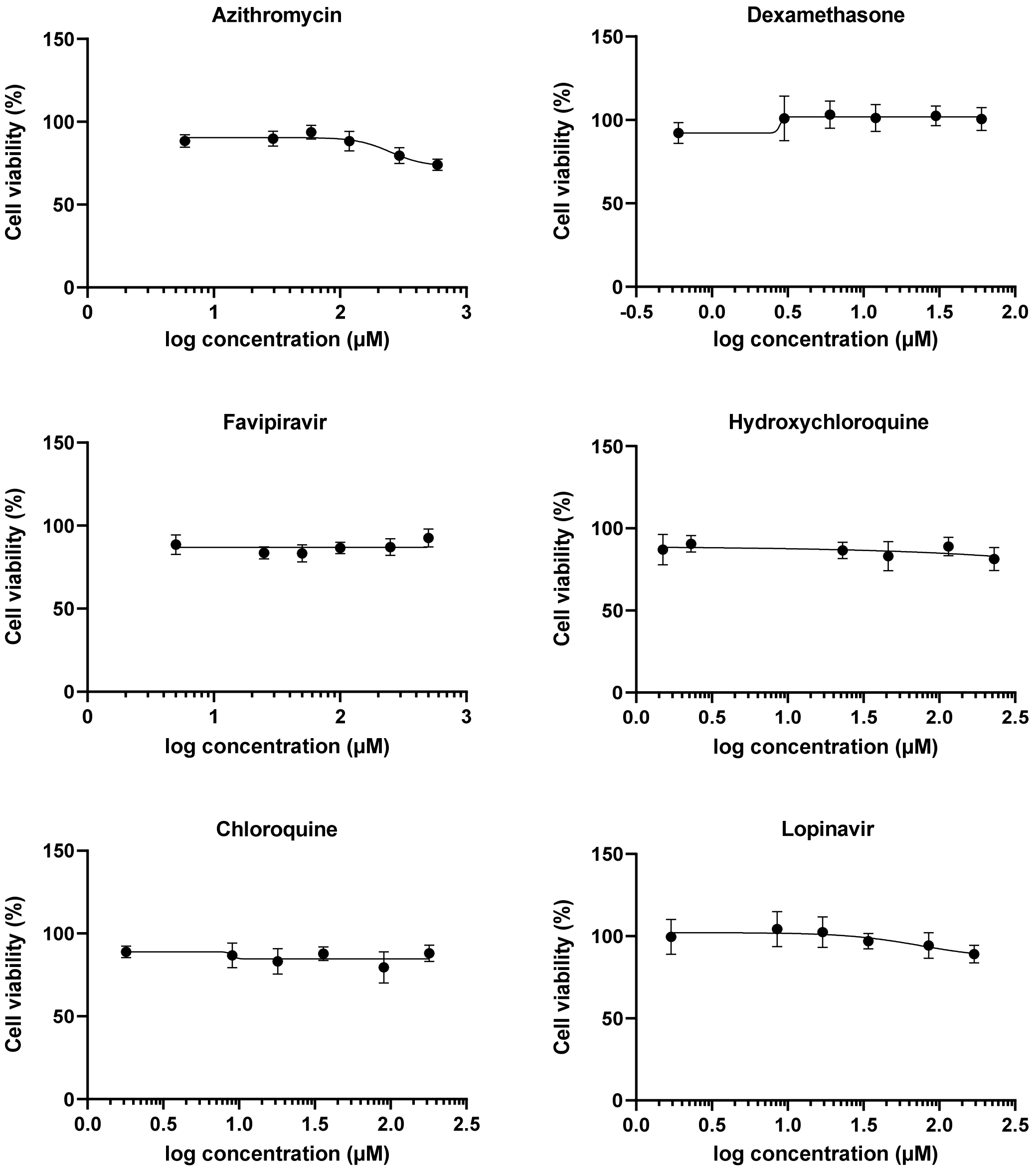

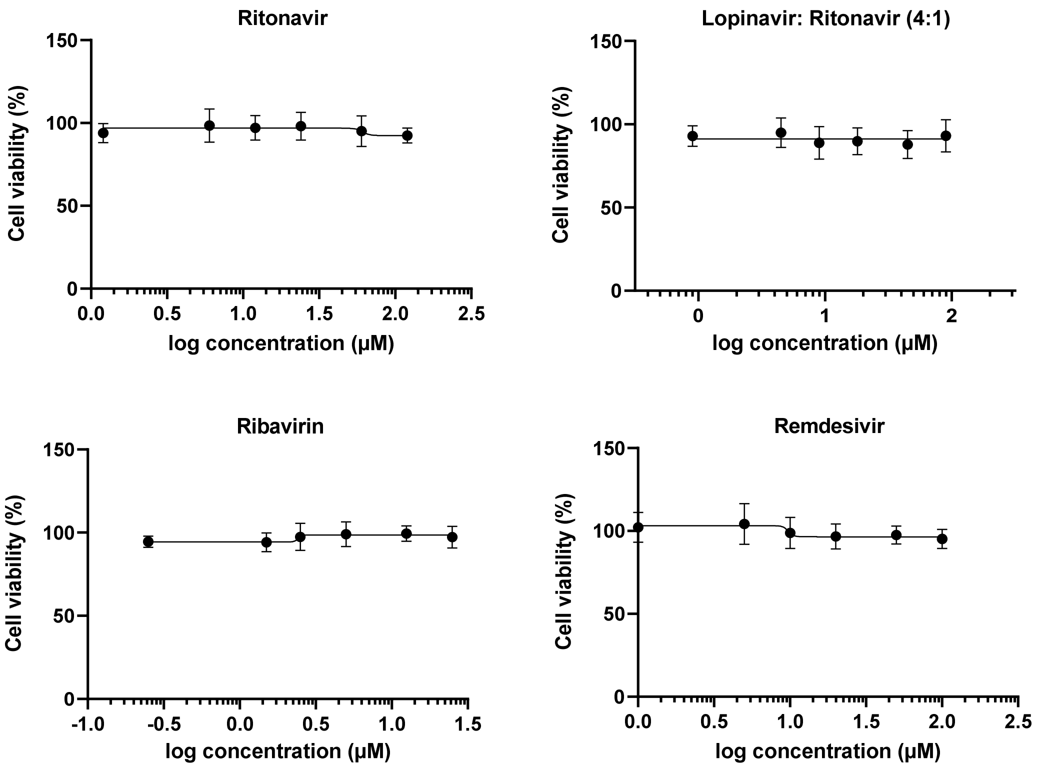

Figure S1. Cell viability curves for the determination of the cytotoxicity levels after 45 minutes exposure of Dubca cells overexpressing human Panx1 to the drug panel. Drugs are categorized as cytotoxic in the concentrations that decreases cell viability with more than 20%. Data are expressed as mean ± standard deviation (*N* = 3, *n* = 4) and visualised in separate graphs per drug.

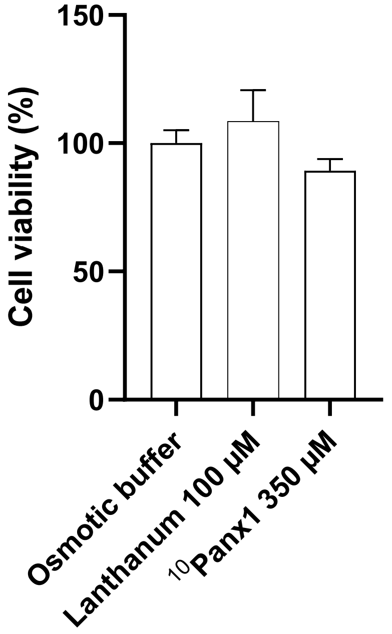

Figure S2. Cell viability after 45 minutes exposure of Dubca cells overexpressing human Panx1 to the positive controls (100 μM of Lanthanum and 350 μM of ^{10}Panx1) in the Panx1 channel activity assay. Drugs are categorized as cytotoxic in the concentrations that decreases cell viability with more than 20%. Data are expressed as mean ± standard deviation (*N* = 3, *n* = 4).

Azithromycin

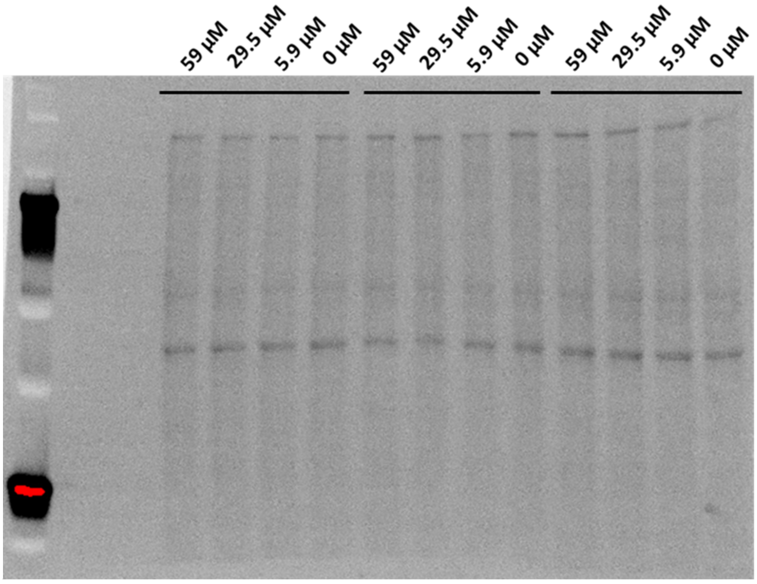

Dexamethasone

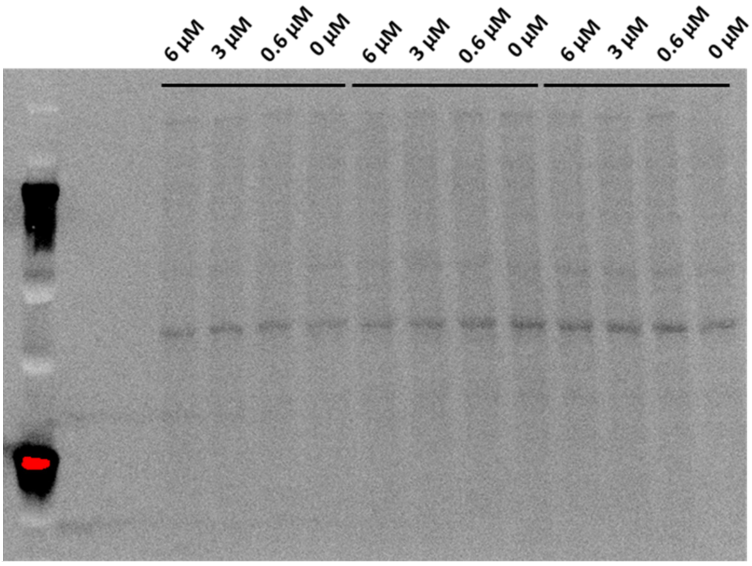

Favipiravir

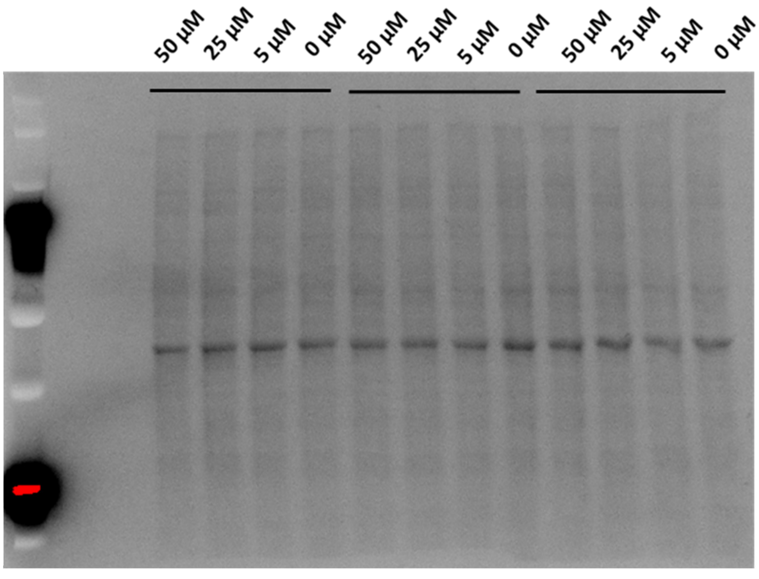

Hydroxychloroquine

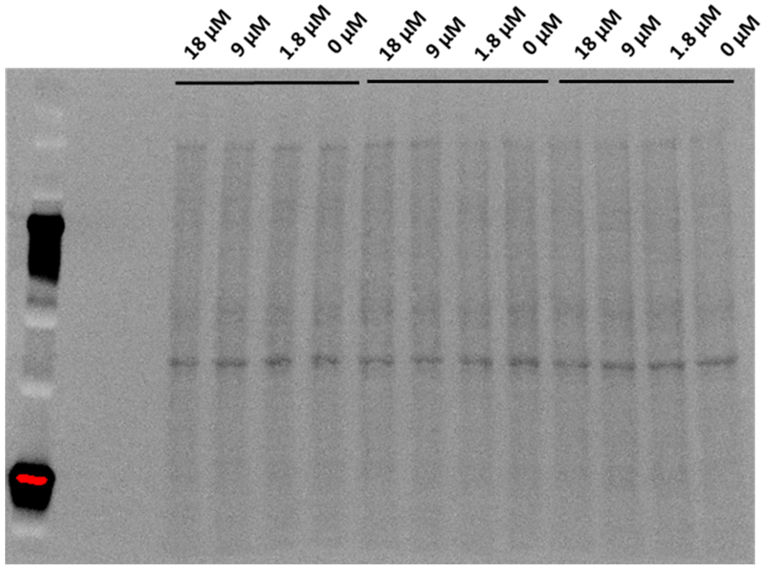

Chloroquine

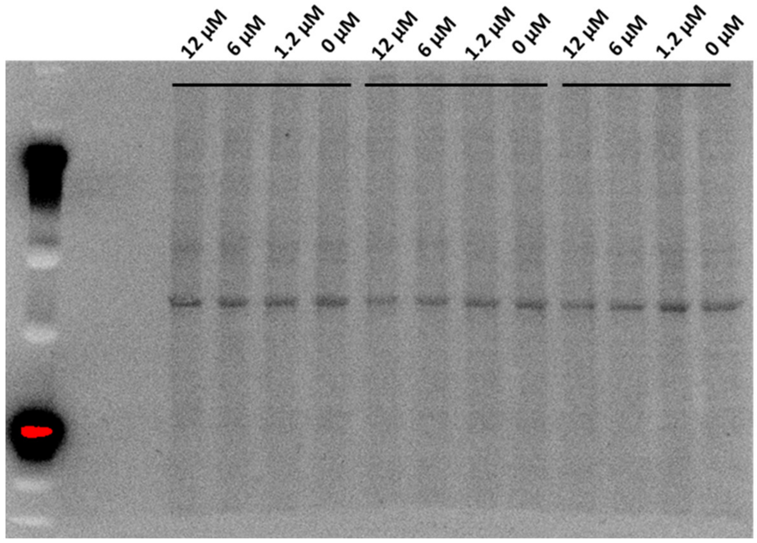

Lopinavir

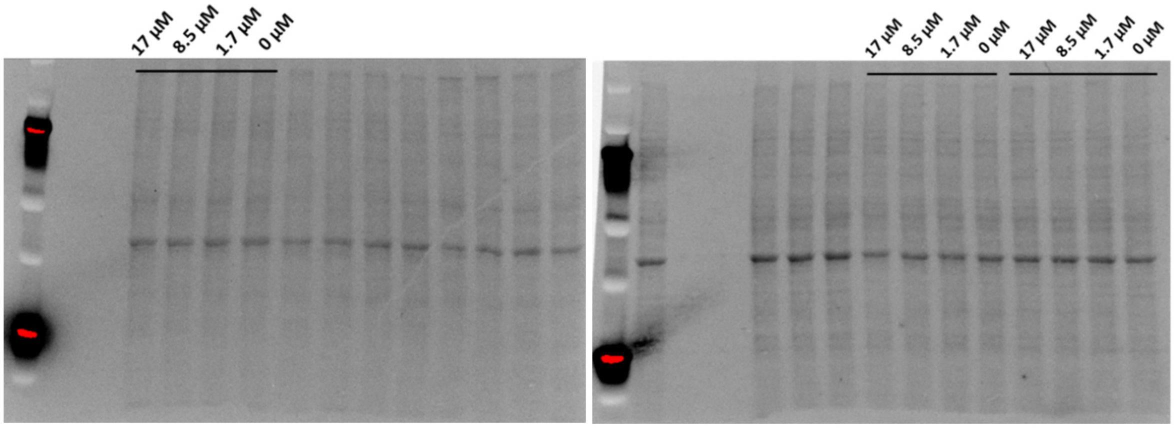

Ritonavir

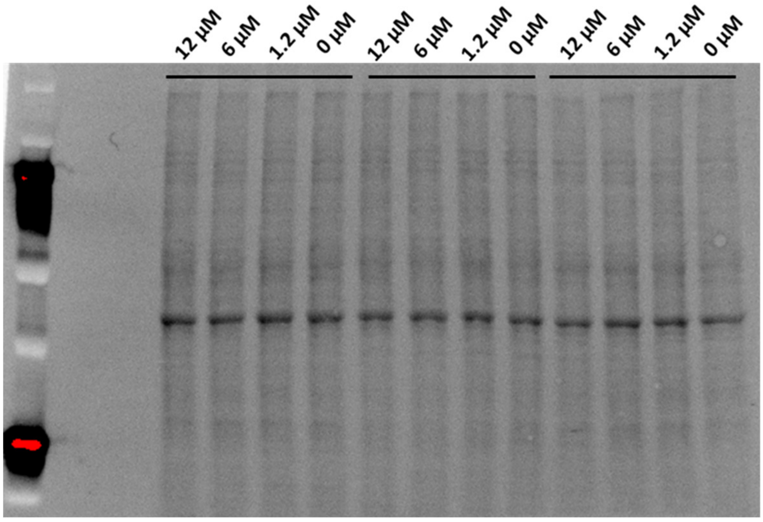

Lopinavir: Ritonavir (4:1)

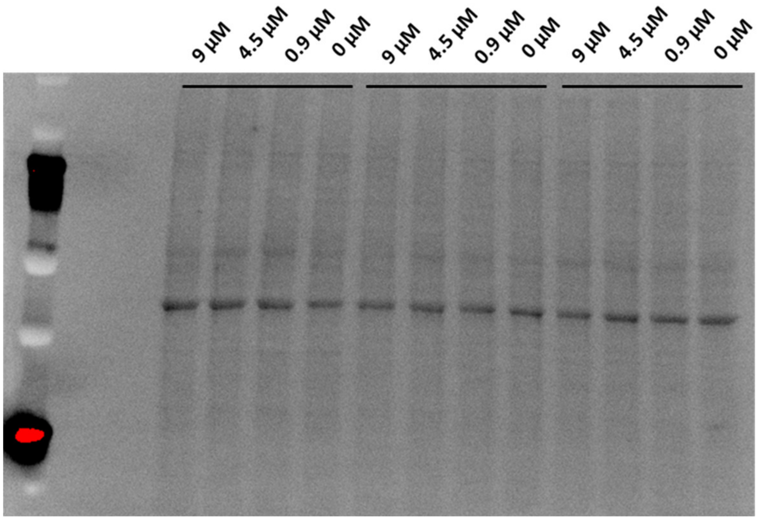

Ribavirin

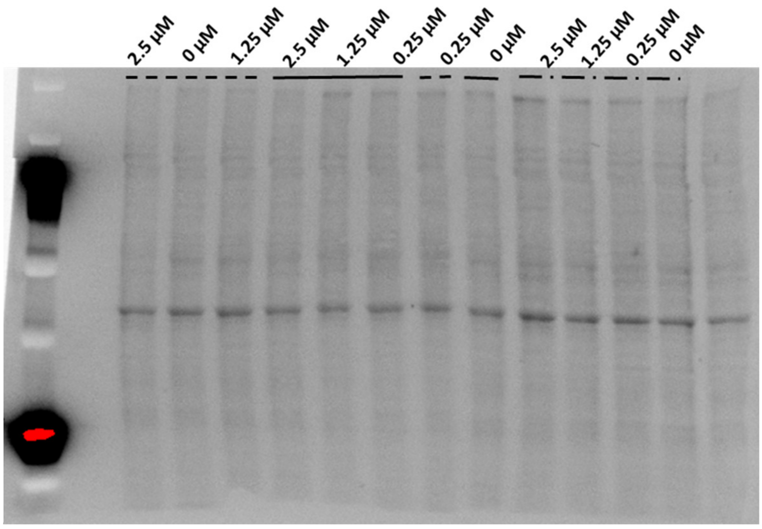

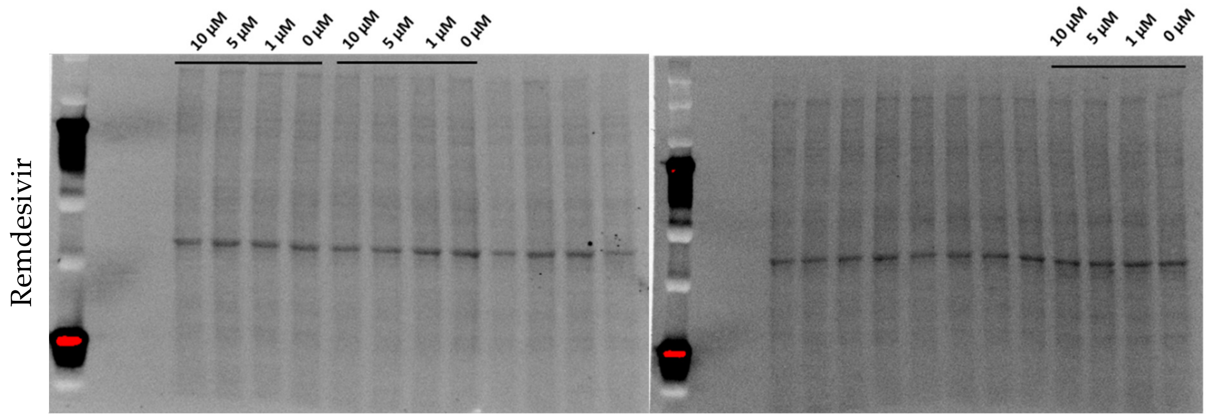

Figure S3. Panx1 total protein loading for normalization of immunoblot analysis. Before incubation with the primary antibody directed against Panx1, total protein loading was visualized on a ChemiDoc™ MP imaging system. Image Lab 6.1 software was used for densiometric analysis. Total protein loading was used to normalize immunoblot signals.

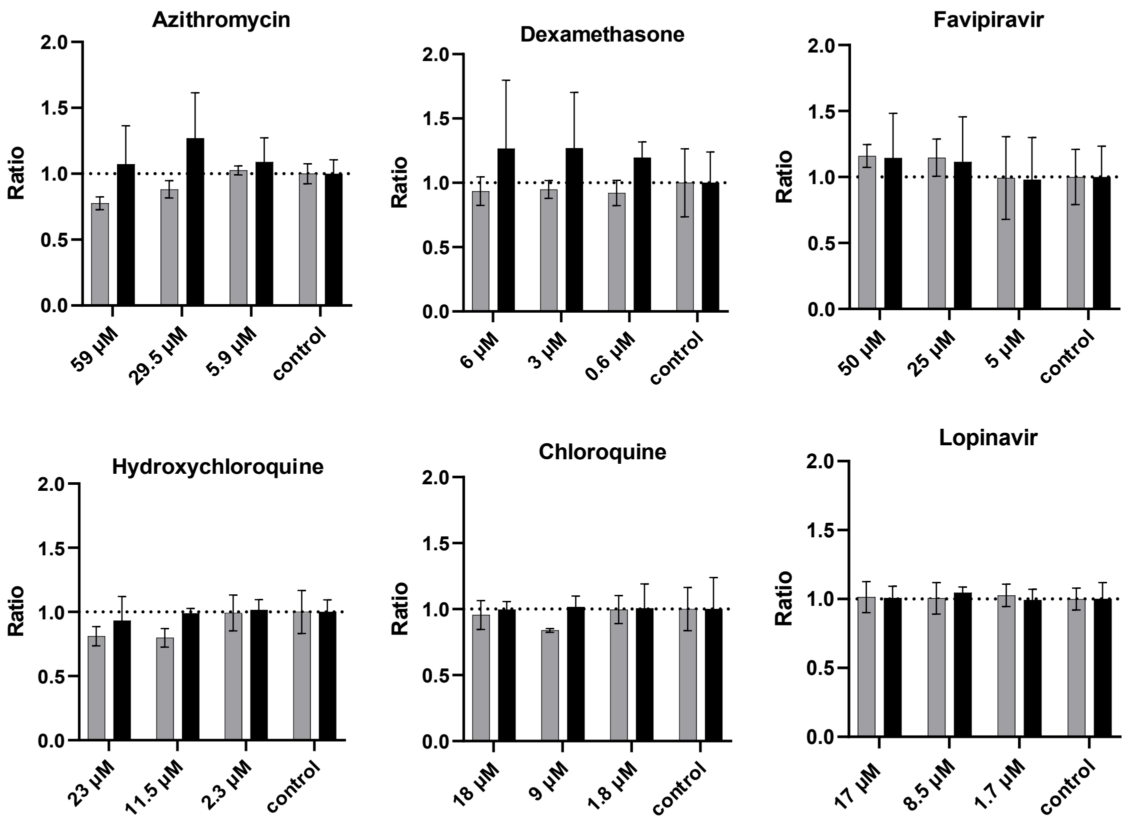

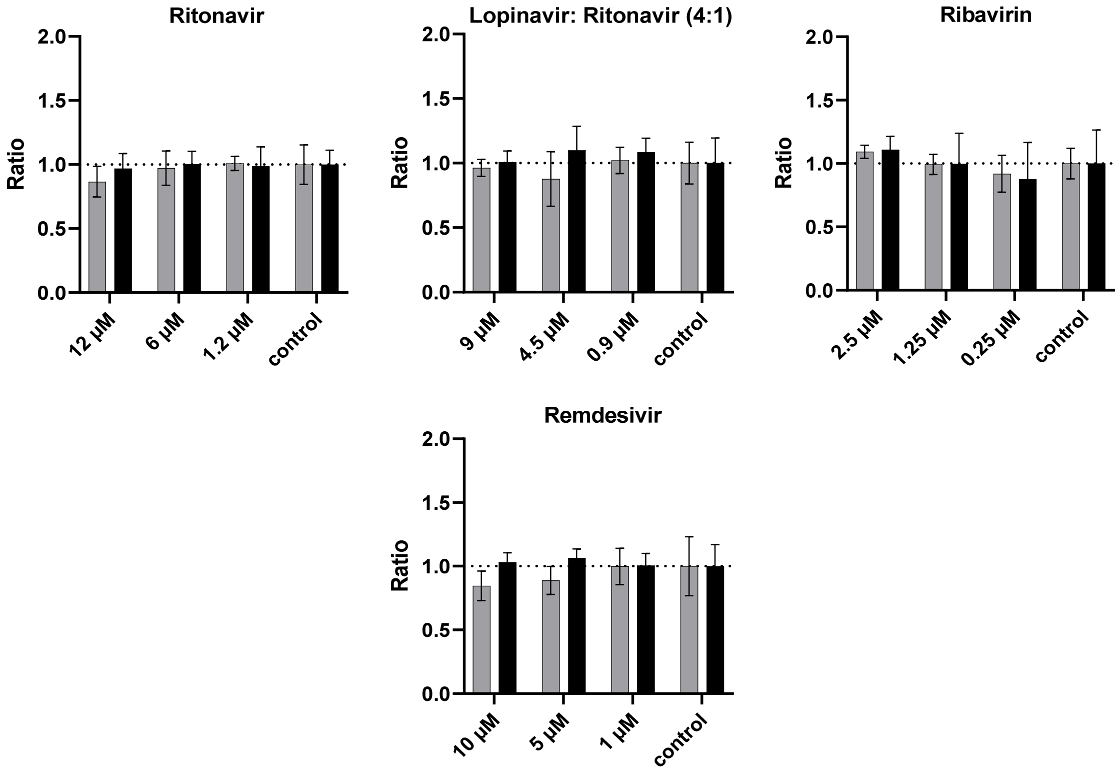

Figure S4. Ratio of the non-glycosylated (Gly0) isoform against the 2 glycosylated isoforms of Panx1 (Gly1 and Gly2). Transduced Dubca cells overexpressing human Panx1 were exposed for 24 h to the drug panel. Immunoblotting was performed and data were extracted from the obtained blots using Image Lab 6.1 software. Afterwards, data were normalized against total protein loading and a ratio of Gly1 and Gly0 (grey bars; Gly1/Gly0) and Gly2 and Gly0 (black bars; Gly2/Gly0) was calculated per drug. The ratios were normalized to the fold change of their respective control. Data are expressed as mean ± standard deviation (N = 3, n = 1) and visualised in separate graphs per drug.
